# Pan-cancer analyses reveal the molecular and clinical characteristics of TET family members and suggests that TET3 maybe a potential therapeutic target

**DOI:** 10.3389/fphar.2024.1418456

**Published:** 2024-07-22

**Authors:** Chunyan Zhang, Jie Zheng, Jin Liu, Yanxia Li, Guoqiang Xing, Shupeng Zhang, Hekai Chen, Jian Wang, Zhijiang Shao, Yongyuan Li, Zhongmin Jiang, Yingzi Pan, Xiaozhi Liu, Ping Xu, Wenhan Wu

**Affiliations:** ^1^ Department of General Surgery, Tianjin Fifth Central Hospital, Tianjin, China; ^2^ Tianjin Key Laboratory of Epigenetics for Organ Development of Premature Infants, Tianjin Fifth Central Hospital, Tianjin, China; ^3^ High Altitude Characteristic Medical Research Institute, Huangnan Tibetan Autonomous Prefecture People’s Hospital, Huangnan Prefecture, Qinghai, China; ^4^ Department of Pathology, Tianjin Fifth Central Hospital, Tianjin, China; ^5^ North China University of Science and Technology, Tangshan, Hebei, China; ^6^ Department of Ophthalmology, Peking University First Hospital, Beijing, China; ^7^ Department of Pharmacy, Tianjin Fifth Central Hospital, Tianjin, China; ^8^ Department of General Surgery, Peking University First Hospital, Beijing, China

**Keywords:** TET family genes, pan-cancer analysis, tumor microenvironment, drug sensitivity, therapeutic target

## Abstract

The Ten-Eleven Translocation (TET) family genes are implicated in a wide array of biological functions across various human cancers. Nonetheless, there is a scarcity of studies that comprehensively analyze the correlation between TET family members and the molecular phenotypes and clinical characteristics of different cancers. Leveraging updated public databases and employing several bioinformatics analysis methods, we assessed the expression levels, somatic variations, methylation levels, and prognostic values of TET family genes. Additionally, we explored the association between the expression of TET family genes and pathway activity, tumor microenvironment (TME), stemness score, immune subtype, clinical staging, and drug sensitivity in pan-cancer. Molecular biology and cytology experiments were conducted to validate the potential role of TET3 in tumor progression. Each TET family gene displayed distinct expression patterns across at least ten detected tumors. The frequency of Single Nucleotide Variant (SNV) in TET genes was found to be 91.24%, primarily comprising missense mutation types, with the main types of copy number variant (CNV) being heterozygous amplifications and deletions. TET1 gene exhibited high methylation levels, whereas TET2 and TET3 genes displayed hypomethylation in most cancers, which correlated closely with patient prognosis. Pathway activity analysis revealed the involvement of TET family genes in multiple signaling pathways, including cell cycle, apoptosis, DNA damage response, hormone AR, PI3K/AKT, and RTK. Furthermore, the expression levels of TET family genes were shown to impact the clinical staging of tumor patients, modulate the sensitivity of chemotherapy drugs, and thereby influence patient prognosis by participating in the regulation of the tumor microenvironment, cellular stemness potential, and immune subtype. Notably, TET3 was identified to promote cancer progression across various tumors, and its silencing was found to inhibit tumor malignancy and enhance chemotherapy sensitivity. These findings shed light on the role of TET family genes in cancer progression and offer insights for further research on TET3 as a potential therapeutic target for pan-cancer.

## Introduction

Epigenetics, including DNA methylation, histone modifications, and non-coding RNA regulation, plays a crucial role in human disease, particularly cancer, by orchestrating gene expression dynamics without altering DNA sequence. Aberrant epigenetic changes, such as hypermethylation of tumor suppressor gene promoters and dysregulated histone modifications, disrupt normal cellular processes, fostering tumor initiation, progression, and metastasis. Despite the continuous advancements in surgical and targeted therapies in recent years, a considerable portion of tumor patients still present with advanced and widespread metastasis at the time of diagnosis, resulting in a bleak prognosis (Golemis et al., 2018). Consequently, conducting comprehensive research into the molecular mechanisms underlying cancer development holds paramount importance for tumor prevention, early screening and diagnosis, clinical treatment, as well as enhancing patient prognosis and survival rates (Alqahtani et al., 2019; Ban et al., 2021; Rodriguez et al., 2022).

Genetic variations and epigenetic changes play pivotal roles in the initiation and progression of tumors (Talib et al., 2021). Nearly all tumors involve single or tandem mutations in one or multiple genes (Kossenas and Constantinou, 2021). To devise precise treatment strategies capable of effectively targeting tumor cells while minimizing damage to normal tissues, from the myriad of small molecule chemical drugs available, a comprehensive understanding of the underlying mechanisms of the implicated genes is imperative (Srivastava and Goodwin, 2020). The advent and advancement of modern bioinformatics methodologies offer a streamlined approach for humanity to efficiently explore and analyze tumor occurrences within established gene information databases, thereby providing guidance for tumor treatment (Nooter and Stoter, 1996; Zaridze, 2008; Gonçalves et al., 2021).

Recent studies have revealed that epigenetic regulations, such as DNA methylation and demethylation modifications, are intricately linked to the onset and progression of human cancers (Kulis and Esteller, 2010; Klutstein, Nejman, Greenfield and Cedar, 2016; Nishiyama and Nakanishi, 2021). DNA methylation often occurs on the CpG islands of the promoter regions, resulting in the activation of oncogenes and the inactivation of tumor suppressor genes (Kulis and Esteller, 2010; Papanicolau-Sengos and Aldape, 2022). Aberrant DNA methylation can disrupt the apoptosis process of cells, render tumor cells insensitive to growth inhibition signals, and induce uncontrolled replication of tumor cells (Pan et al., 2018). Consequently, epigenetic research, particularly focusing on DNA methylation, holds significant value for tumor molecular diagnosis, prevention, and treatment, as well as predicting therapeutic outcomes and prognosis (C. Chen et al., 2022; Meng et al., 2015).

Studies indicate that both hypermethylation and hypomethylation play crucial roles in tumor development. The Ten-Eleven Translocation (TET) enzymes facilitate the oxidation of 5-methylcytosines (5mCs) and promote site-specific reversal of DNA methylation (Kohli and Zhang, 2013). TET family genes, including TET1, TET2, and TET3, exhibit diverse vital biological functions in mammals (Kinney and Pradhan, 2013; Zeng and Chen, 2019; Joshi, Liu, Breslin and Zhang, 2022). At their core, TET family genes consist of α-Ketoglutaric acid (α-KG) and Fe2+-dependent dioxygenases, which regulate DNA demethylation, modulate gene transcription, and influence various life processes such as embryonic development, as well as the onset of various diseases including tumors (Rasmussen and Helin, 2016; Ma et al., 2021). Additionally, studies have demonstrated that TET family genes not only facilitate active DNA demethylation but also impede methylation propagation by maintaining a low DNA methylation state, thus closely associating them with tumorigenesis (Shekhawat et al., 2021). Notably, TET family genes exhibit a dual role in different tumors, displaying both carcinogenic and anticancer effects.

While TET family genes are known to play an indispensable role in the progression of numerous tumors, there is currently scarce literature on pan-cancer analysis of these genes. Therefore, in this study, we conducted an analysis of the relationship between TET family genes and pan-cancer, exploring various aspects including mRNA expression levels, somatic cell variations, DNA methylation patterns, pathway activities, survival rates and prognoses, immune subtypes specific to individual tumors, tumor microenvironment (TME), stem cell indices, and drug sensitivities. Our aim is to provide valuable reference data and constructive suggestions based on a comprehensive understanding of the landscape.

## Results

### Expression and differential analysis of TET family genes in pan-cancer

**FIGURE 1:**
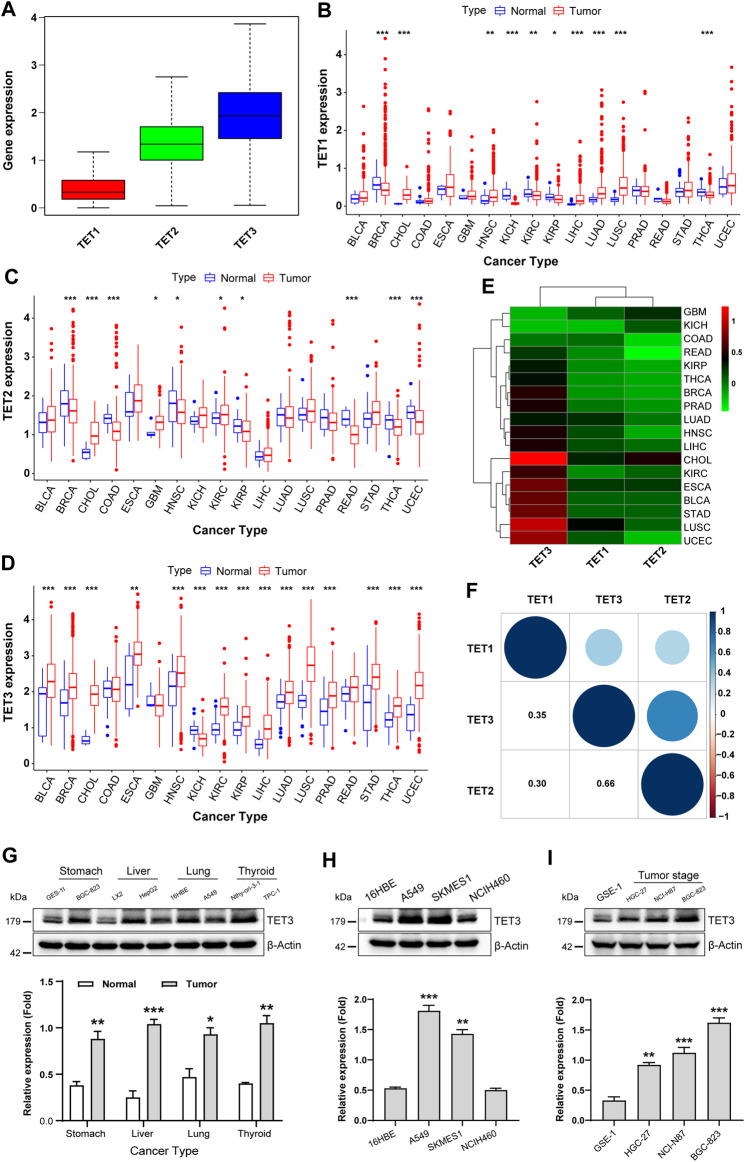
Expression and differential analysis of TET family genes in pan-cancer. **(A)** TET1, TET2, and TET3 expression was assessed across pan-cancer samples. **(B–D)** Differences in TET1-3 expression between pan-cancer and normal tissues were analyzed. **(E)** A heatmap visualized expression disparities among tumors using Wilcox analysis. **(F)** The expression correlation among TET family genes was analyzed in pan-cancer. **(G–I)** TET3 protein expression was evaluated across various cancer cell types **(G)**, lung tumor subtypes **(H)**, and gastric cancer cell differentiation levels **(I)**. Data are shown as mean ± SD, with statistical significance denoted as *p < 0.05, **p < 0.01, ***p < 0.001.

Firstly, utilizing transcriptome data from 33 tumors available in the UCSC Xena database, we conducted an extraction and analysis of the expression profiles of the target genes belonging to the TET family (specifically, TET1, TET2, and TET3) across various types of cancer. The results depicted in the Boxplot revealed that the expression level of the TET3 gene ranked highest across pan-cancer samples, followed by TET2, with TET1 exhibiting the lowest expression (Figure 1A, Supplementary Table S1).

Next, we screened 18 types of tumors, each containing at least five corresponding normal samples, and analyzed the expression differences of TET family members in pan-cancer and its normal tissues. As shown in Figure 1B, TET1 exhibited abnormal expression in 10 types of tumors, with significantly downregulated expression observed in BRCA, KICH, KIRC, KIRP, and THCA, and upregulated expression noted in CHOL, HNSC, LIHC, LUAD, and LUSC tumors compared to their corresponding normal samples. The expression of TET2, in tumor samples such as BRCA, COAD, HNSC, KIRP, READ, and THCA, was lower, while in CHOL, GBM, KIRC, and UCEC tumors, it was significantly higher than that in corresponding normal control tissues (Figure 1C). The expression of the TET3 gene varied across 15 tumors and corresponding normal samples, including BLCA, BRCA, CHOL, ESCA, HNSC, KIRC, KIRP, LIHC, LUAD, LUSC, PRAD, STAD, THCA, UCEC, and KICH. Among these, except for KICH, TET3 was significantly overexpressed in all 14 other tumors (Figure 1D). These findings suggest that the high expression of TET3 may serve as a key factor in the occurrence, development, and poor prognosis of various clinical tumors.

To further elucidate the differential expression patterns of TET family members in various tumors, we conducted Wilcox analysis on the expression differences in each tumor and obtained the aforementioned differential expression cluster analysis graph (Figure 1E; Table 1). As previously mentioned, TET3 was significantly upregulated, while TET1 and TET2 were downregulated in most tumors. It remains unclear whether there is a correlation between the expression of the three members of the TET family in pan-cancer. Therefore, we conducted a detailed analysis of the correlation between TET family genes in pan-cancer. As illustrated in Figure 1F, the expression of TET1, TET2, and TET3 positively correlated in pan-cancer, with a stronger positive correlation observed between TET2 and TET3.

**TABLE 1:** Differential expression of TET family members in various tumors.

CancerType	TET1	TET2	TET3
BLCA	0.096236305	0.101847392	0.716234375
BRCA	−0.065518523	−0.144760041	0.506621202
CHOL	0.283859336	0.49181886	1.242806426
COAD	0.065707243	−0.283057513	0.027092671
ESCA	0.211017314	0.209220152	0.772441549
GBM	0.075892311	0.246159584	−0.18453126
HNSC	0.140153387	−0.137649947	0.479021335
KICH	−0.222646071	0.105693609	−0.22620435
KIRC	−0.030193499	0.079717096	0.589176718
KIRP	−0.044008843	−0.153032274	0.3255915
LIHC	0.165642026	0.057092953	0.501816772
LUAD	0.261827716	0.026681648	0.315641285
LUSC	0.399485757	0.093571817	1.013154683
PRAD	0.016403461	−0.083845777	0.483879586
READ	−0.039904543	−0.431725117	0.209207574
STAD	0.078809067	0.089785518	0.754909001
THCA	−0.066662138	−0.14246154	0.363362479
UCEC	0.121399047	−0.207671334	0.881939819

Given the high expression of TET3 in numerous tumors, we speculate that it may represent a potential therapeutic target. Consequently, we assessed the expression of TET3 protein in four tumor cell lines (BGC-823, HepG2, A549, TPC-1) and their corresponding normal cell lines (GES-1, LX2, 16HBE, Nthy-ori-3-1). The results demonstrated higher expression levels of TET3 in all four tumor cell lines compared to the corresponding control cell lines (Figures 1G–I). Subsequently, we examined the expression of TET3 protein in three different pathological subtypes of lung cancer cell lines (A549, SKMES1, NCIH460). The findings revealed significantly higher expression levels of TET3 protein in lung adenocarcinoma A549 and lung squamous cell carcinoma SKMES1 compared to normal controls and large cell lung cancer cell lines. This observation suggests differential expression of TET3 protein within the same type of tumors with varying pathological subtypes. Finally, we evaluated the expression of TET3 protein in gastric cancer cell lines (NCI-N87, BGC-823, HGC-27) with differing degrees of differentiation. The results indicated that as the degree of cell differentiation decreased, the expression of TET3 protein gradually increased. This implies that the expression intensity of TET3 protein may serve as an indicator of the malignant level of gastric cancer.

### Somatic cell variation landscape of TET family genes

To comprehend the genomic alterations of TET1-3 in tumors, we scrutinized single nucleotide variant data (SNV, Table 2) and copy number variant data (CNV, Table 3) from a total of 10,289 pan-cancer samples spanning 33 types of tumors, and illustrated SNV and CNV landscapes.

**TABLE 2:** The single nucleotide variants index of TETs in the tumor genome.

Cancertype	Symbol	Effective Mut	Non Effective Mut	sample_size	Percentage	Entrez
ACC	TET1	2	1	92	2.173913043	80312
ACC	TET2	1	3	92	1.086956522	54790
ACC	TET3	1	1	92	1.086956522	200424
BLCA	TET1	26	8	411	6.326034063	80312
BLCA	TET2	9	4	411	2.189781022	54790
BLCA	TET3	11	4	411	2.676399027	200424
BRCA	TET1	14	11	1026	1.364522417	80312
BRCA	TET2	14	5	1026	1.364522417	54790
BRCA	TET3	12	6	1026	1.169590643	200424
CESC	TET1	11	3	291	3.780068729	80312
CESC	TET2	6	15	291	2.06185567	54790
CESC	TET3	8	3	291	2.749140893	200424
CHOL	TET2	0	1	36	0	54790
CHOL	TET3	1	0	36	2.777777778	200424
COAD	TET1	26	10	407	6.388206388	80312
COAD	TET2	19	30	407	4.668304668	54790
COAD	TET3	26	9	407	6.388206388	200424
DLBC	TET1	1	1	37	2.702702703	80312
DLBC	TET2	3	1	37	8.108108108	54790
DLBC	TET3	0	1	37	0	200424
ESCA	TET1	4	3	185	2.162162162	80312
ESCA	TET2	4	5	185	2.162162162	54790
ESCA	TET3	3	4	185	1.621621622	200424
GBM	TET1	8	2	403	1.985111663	80312
GBM	TET2	4	2	403	0.992555831	54790
GBM	TET3	5	1	403	1.240694789	200424
HNSC	TET1	9	4	509	1.768172888	80312
HNSC	TET2	7	5	509	1.37524558	54790
HNSC	TET3	9	1	509	1.768172888	200424
KICH	TET1	1	0	66	1.515151515	80312
KICH	TET2	0	1	66	0	54790
KICH	TET3	0	1	66	0	200424
KIRC	TET1	3	3	370	0.810810811	80312
KIRC	TET2	5	0	370	1.351351351	54790
KIRC	TET3	2	1	370	0.540540541	200424
KIRP	TET1	7	0	282	2.482269504	80312
KIRP	TET2	2	2	282	0.709219858	54790
KIRP	TET3	2	2	282	0.709219858	200424
LAML	TET1	1	0	85	1.176470588	80312
LGG	TET1	2	1	526	0.380228137	80312
LGG	TET2	5	2	526	0.950570342	54790
LGG	TET3	1	2	526	0.190114068	200424
LIHC	TET1	7	0	365	1.917808219	80312
LIHC	TET2	2	11	365	0.547945205	54790
LIHC	TET3	4	0	365	1.095890411	200424
LUAD	TET1	25	7	567	4.409171076	80312
LUAD	TET2	11	3	567	1.940035273	54790
LUAD	TET3	13	3	567	2.292768959	200424
LUSC	TET1	20	7	485	4.12371134	80312
LUSC	TET2	19	1	485	3.917525773	54790
LUSC	TET3	14	6	485	2.886597938	200424
MESO	TET1	1	0	82	1.219512195	80312
MESO	TET2	0	1	82	0	54790
OV	TET1	6	3	412	1.45631068	80312
OV	TET2	1	1	412	0.242718447	54790
OV	TET3	7	1	412	1.699029126	200424
PAAD	TET1	1	0	178	0.561797753	80312
PAAD	TET2	1	0	178	0.561797753	54790
PAAD	TET3	2	0	178	1.123595506	200424
PRAD	TET1	4	1	498	0.803212851	80312
PRAD	TET2	1	1	498	0.200803213	54790
PRAD	TET3	4	1	498	0.803212851	200424
READ	TET1	8	3	149	5.369127517	80312
READ	TET2	4	7	149	2.684563758	54790
READ	TET3	4	3	149	2.684563758	200424
SARC	TET1	2	3	239	0.836820084	80312
SARC	TET2	6	1	239	2.510460251	54790
SARC	TET3	3	2	239	1.255230126	200424
SKCM	TET1	52	21	468	11.111111111	80312
SKCM	TET2	37	14	468	7.905982906	54790
SKCM	TET3	42	25	468	8.974358974	200424
STAD	TET1	22	8	439	5.011389522	80312
STAD	TET2	17	2	439	3.872437358	54790
STAD	TET3	21	8	439	4.783599089	200424
TGCT	TET1	4	1	151	2.649006623	80312
THCA	TET1	2	0	500	0.4	80312
THCA	TET2	1	0	500	0.2	54790
THCA	TET3	2	2	500	0.4	200424
THYM	TET1	1	1	123	0.81300813	80312
THYM	TET2	1	0	123	0.81300813	54790
THYM	TET3	1	0	123	0.81300813	200424
UCEC	TET1	57	29	531	10.734463277	80312
UCEC	TET2	46	45	531	8.662900188	54790
UCEC	TET3	51	43	531	9.604519774	200424
UCS	TET1	1	1	57	1.754385965	80312
UCS	TET2	1	1	57	1.754385965	54790
UCS	TET3	1	0	57	1.754385965	200424
UVM	TET3	1	0	80	1.25	200424

**TABLE 3:** The copy number variant index of TETs in the tumor genome.

Cancertype	Symbol	a_total	d_total	a_hete	d_hete	a_homo	d_homo	Entrez
ACC	TET1	27.7777778	13.3333333	27.7777778	13.3333333	0	0	80312
ACC	TET2	38.8888889	12.2222222	38.8888889	12.2222222	0	0	54790
ACC	TET3	15.5555556	20	15.5555556	18.8888889	0	1.1111111	200424
BLCA	TET1	10.7843137	35.0490196	9.8039216	33.8235294	0.9803922	1.2254902	80312
BLCA	TET2	7.5980392	37.0098039	7.1078431	36.7647059	0.4901961	0.245098	54790
BLCA	TET3	33.0882353	8.0882353	31.6176471	8.0882353	1.4705882	0	200424
BRCA	TET1	12.5925926	21.7592593	12.037037	21.5740741	0.5555556	0.1851852	80312
BRCA	TET2	9.537037	29.4444444	9.0740741	29.0740741	0.462963	0.3703704	54790
BRCA	TET3	16.2962963	15	15.7407407	14.9074074	0.5555556	0.0925926	200424
CESC	TET1	5.7627119	25.0847458	5.7627119	25.0847458	0	0	80312
CESC	TET2	6.1016949	32.2033898	6.1016949	31.8644068	0	0.3389831	54790
CESC	TET3	23.0508475	5.7627119	23.0508475	5.7627119	0	0	200424
CHOL	TET1	16.6666667	13.8888889	11.1111111	13.8888889	5.5555556	0	80312
CHOL	TET2	5.5555556	44.4444444	5.5555556	44.4444444	0	0	54790
CHOL	TET3	11.1111111	5.5555556	11.1111111	5.5555556	0	0	200424
COAD	TET1	5.5432373	19.5121951	5.3215078	19.5121951	0.2217295	0	80312
COAD	TET2	3.7694013	29.9334812	3.7694013	29.7117517	0	0.2217295	54790
COAD	TET3	17.9600887	1.5521064	17.9600887	1.5521064	0	0	200424
DLBC	TET1	10.4166667	6.25	10.4166667	6.25	0	0	80312
DLBC	TET2	0	10.4166667	0	10.4166667	0	0	54790
DLBC	TET3	12.5	6.25	10.4166667	2.0833333	2.0833333	4.1666667	200424
ESCA	TET1	19.5652174	27.7173913	18.4782609	27.7173913	1.0869565	0	80312
ESCA	TET2	10.8695652	52.7173913	10.8695652	52.7173913	0	0	54790
ESCA	TET3	39.673913	4.3478261	38.5869565	4.3478261	1.0869565	0	200424
GBM	TET1	0.3466205	87.3483536	0.3466205	86.8284229	0	0.5199307	80312
GBM	TET2	4.6793761	10.745234	4.6793761	10.5719237	0	0.1733102	54790
GBM	TET3	6.2391681	6.7590988	6.2391681	6.7590988	0	0	200424
HNSC	TET1	8.2375479	23.9463602	7.6628352	23.9463602	0.5747126	0	80312
HNSC	TET2	9.3869732	30.4597701	9.3869732	30.2681992	0	0.1915709	54790
HNSC	TET3	21.2643678	4.789272	20.8812261	4.789272	0.3831418	0	200424
KICH	TET1	4.5454545	72.7272727	4.5454545	72.7272727	0	0	80312
KICH	TET2	34.8484848	1.5151515	34.8484848	1.5151515	0	0	54790
KICH	TET3	3.030303	69.6969697	3.030303	69.6969697	0	0	200424
KIRC	TET1	2.2727273	16.8560606	2.2727273	16.8560606	0	0	80312
KIRC	TET2	2.2727273	14.2045455	2.0833333	14.0151515	0.1893939	0.1893939	54790
KIRC	TET3	14.3939394	2.6515152	14.3939394	2.6515152	0	0	200424
KIRP	TET1	2.4305556	5.9027778	2.4305556	5.9027778	0	0	80312
KIRP	TET2	3.8194444	9.0277778	3.8194444	9.0277778	0	0	54790
KIRP	TET3	14.9305556	2.4305556	14.9305556	2.0833333	0	0.3472222	200424
LAML	TET1	1.5706806	0.5235602	1.5706806	0.5235602	0	0	80312
LAML	TET2	2.0942408	1.5706806	2.0942408	1.0471204	0	0.5235602	54790
LAML	TET3	1.0471204	0	1.0471204	0	0	0	200424
LGG	TET1	0.7797271	19.6881092	0.7797271	19.6881092	0	0	80312
LGG	TET2	1.1695906	20.0779727	0.7797271	19.6881092	0.3898635	0.3898635	54790
LGG	TET3	2.5341131	4.288499	2.5341131	4.288499	0	0	200424
LIHC	TET1	11.8918919	21.0810811	11.3513514	21.0810811	0.5405405	0	80312
LIHC	TET2	1.8918919	47.027027	1.8918919	46.2162162	0	0.8108108	54790
LIHC	TET3	11.8918919	10	11.8918919	10	0	0	200424
LUAD	TET1	15.8914729	25.5813953	15.1162791	25.1937984	0.7751938	0.3875969	80312
LUAD	TET2	9.6899225	31.3953488	9.496124	31.3953488	0.1937984	0	54790
LUAD	TET3	28.875969	5.2325581	28.2945736	5.2325581	0.5813953	0	200424
LUSC	TET1	12.1756487	44.510978	11.7764471	43.7125749	0.3992016	0.7984032	80312
LUSC	TET2	5.5888224	60.2794411	5.3892216	60.0798403	0.1996008	0.1996008	54790
LUSC	TET3	48.3033932	2.1956088	46.3073852	2.1956088	1.996008	0	200424
MESO	TET1	3.4482759	19.5402299	2.2988506	19.5402299	1.1494253	0	80312
MESO	TET2	1.1494253	44.8275862	1.1494253	44.8275862	0	0	54790
MESO	TET3	5.7471264	8.045977	5.7471264	8.045977	0	0	200424
OV	TET1	24.5250432	26.2521589	22.4525043	25.9067358	2.0725389	0.3454231	80312
OV	TET2	4.6632124	72.193437	4.3177893	70.984456	0.3454231	1.208981	54790
OV	TET3	35.4058722	10.1899827	33.5060449	10.0172712	1.8998273	0.1727116	200424
PAAD	TET1	7.6086957	15.2173913	7.0652174	15.2173913	0.5434783	0	80312
PAAD	TET2	5.4347826	15.7608696	5.4347826	15.7608696	0	0	54790
PAAD	TET3	7.6086957	5.9782609	7.6086957	5.9782609	0	0	200424
PCPG	TET1	6.1728395	0.617284	6.1728395	0.617284	0	0	80312
PCPG	TET2	3.0864198	6.1728395	1.8518519	6.1728395	1.2345679	0	54790
PCPG	TET3	3.0864198	6.7901235	3.0864198	6.7901235	0	0	200424
PRAD	TET1	4.4715447	10.5691057	4.0650407	9.7560976	0.4065041	0.8130081	80312
PRAD	TET2	2.4390244	6.300813	2.4390244	4.6747967	0	1.6260163	54790
PRAD	TET3	3.0487805	7.5203252	2.8455285	6.300813	0.203252	1.2195122	200424
READ	TET1	5.4545455	22.4242424	4.8484848	22.4242424	0.6060606	0	80312
READ	TET2	3.030303	44.2424242	3.030303	43.6363636	0	0.6060606	54790
READ	TET3	23.030303	7.2727273	23.030303	7.2727273	0	0	200424
SARC	TET1	3.8910506	56.4202335	3.5019455	55.2529183	0.3891051	1.1673152	80312
SARC	TET2	15.1750973	24.9027237	14.7859922	24.9027237	0.3891051	0	54790
SARC	TET3	12.4513619	32.6848249	11.6731518	32.6848249	0.7782101	0	200424
SKCM	TET1	1.0899183	60.4904632	1.0899183	60.4904632	0	0	80312
SKCM	TET2	12.5340599	26.1580381	11.9891008	25.8855586	0.5449591	0.2724796	54790
SKCM	TET3	17.7111717	13.6239782	17.4386921	13.6239782	0.2724796	0	200424
STAD	TET1	18.8208617	16.7800454	17.4603175	16.3265306	1.3605442	0.4535147	80312
STAD	TET2	4.7619048	39.2290249	4.7619048	38.5487528	0	0.6802721	54790
STAD	TET3	20.4081633	4.0816327	20.1814059	4.0816327	0.2267574	0	200424
TGCT	TET1	4	52	4	52	0	0	80312
TGCT	TET2	0	73.3333333	0	73.3333333	0	0	54790
TGCT	TET3	32.6666667	6	32.6666667	6	0	0	200424
THCA	TET1	0.4008016	2.2044088	0.4008016	1.4028056	0	0.8016032	80312
THCA	TET2	1.002004	0.4008016	1.002004	0.4008016	0	0	54790
THCA	TET3	0.4008016	1.8036072	0.4008016	1.8036072	0	0	200424
THYM	TET1	1.6260163	1.6260163	1.6260163	1.6260163	0	0	80312
THYM	TET2	1.6260163	3.2520325	1.6260163	3.2520325	0	0	54790
THYM	TET3	0.8130081	0.8130081	0.8130081	0.8130081	0	0	200424
UCEC	TET1	22.4489796	10.0185529	21.5213358	10.0185529	0.9276438	0	80312
UCEC	TET2	1.8552876	20.9647495	1.6697588	20.593692	0.1855288	0.3710575	54790
UCEC	TET3	20.0371058	2.0408163	18.3673469	1.8552876	1.6697588	0.1855288	200424
UCS	TET1	39.2857143	33.9285714	39.2857143	33.9285714	0	0	80312
UCS	TET2	3.5714286	69.6428571	3.5714286	69.6428571	0	0	54790
UCS	TET3	44.6428571	5.3571429	44.6428571	5.3571429	0	0	200424
UVM	TET1	0	1.25	0	1.25	0	0	80312
UVM	TET2	5	5	5	5	0	0	54790
UVM	TET3	12.5	0	12.5	0	0	0	200424

**FIGURE 2:**
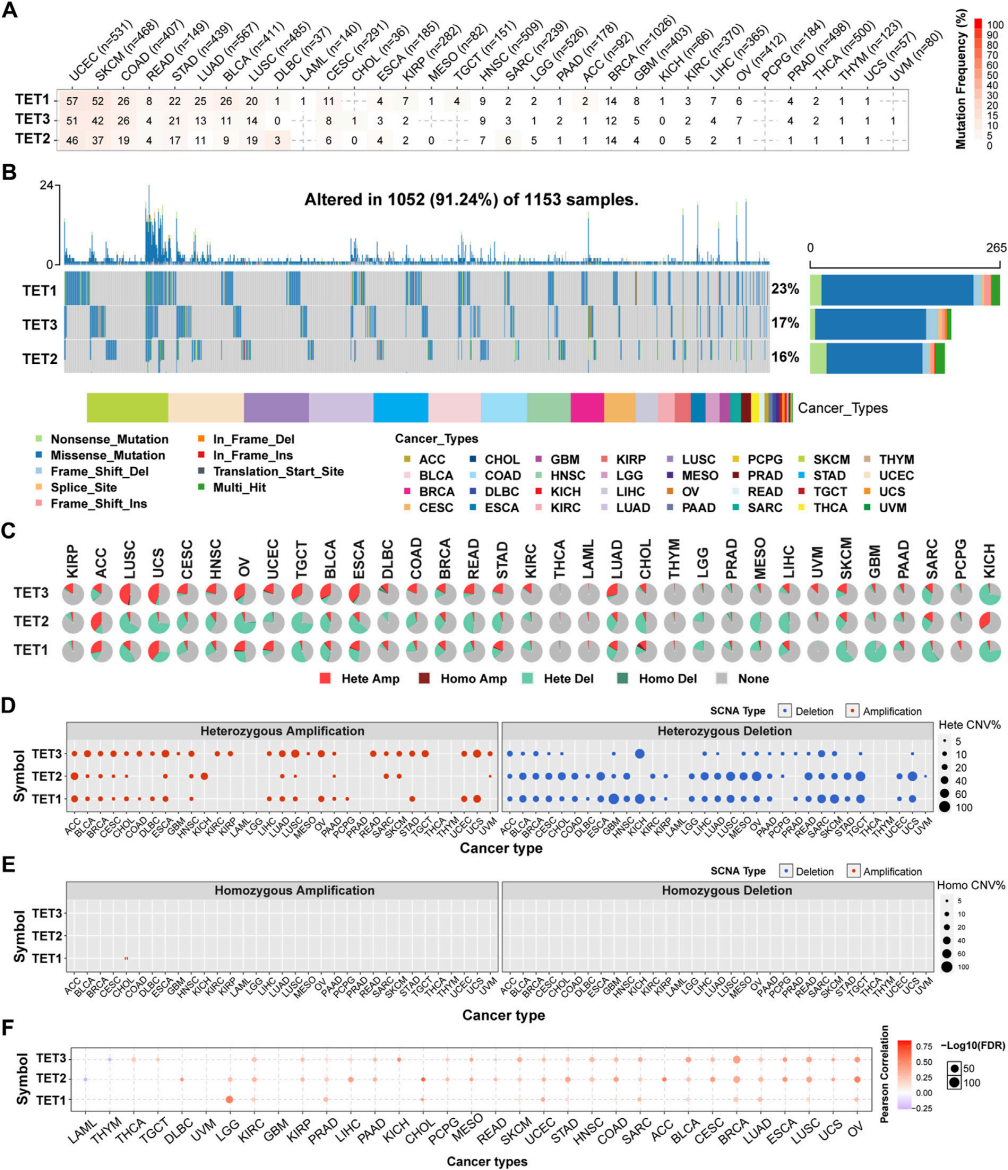
Somatic mutations in TET family genes were analyzed. **(A)** Single Nucleotide Variants (SNV) frequencies of TETs across cancers are presented in a table, indicating mutated gene counts per cancer type. ‘0’ indicates that there was no mutation in the gene coding region, and no number indicates there was no mutation in any region of the gene. **(B)** SNV variant types of TETs illustrate mutation distribution and categorization. **(C)** copy number variant (CNV) distribution across 33 cancers is depicted in pie charts, showing combined heterozygous/homozygous CNV proportions for each gene in each cancer. Hete Amp = heterozygous amplification; Hete Del = heterozygous deletion; Homo Amp = homozygous amplification; Homo Del = homozygous deletion; None = no CNV. **(D, E)** Heterozygous and homozygous CNV profiles display the percentage of CNV, including amplification and deletion, for each gene in each cancer. Only genes with >5% CNV in a given cancer are shown as a point on figure. **(F)** CNV correlation with mRNA expression is depicted through Person’s correlation analysis, with point size indicating statistical significance. FDR, false discovery rate.

Initially, we analyzed the SNP data pertaining to the TET gene to ascertain the frequency and variant types across each cancer subtype. As depicted in Figures 2A, B, among these cancers, the SNV frequency of UCEC, SKCM, and COAD ranges from 19% to 57%. The SNV frequency of the TETs gene collectively is 91.24% (1052/1153). Variant analysis unveiled missense mutation as the predominant SNP type. SNV percentage analysis revealed mutation rates of 23%, 17%, and 16% for TET1-3, respectively.

To elucidate the mutation pattern of CNV, we examined the CNV data of TETs genes in the TCGA database. The distribution of CNV pie charts illustrated heterozygous amplification and heterozygous deletion as the primary types (Figure 2C). CNV percentage analysis unveiled heterozygous amplification rates of TET1 in ACC and UCS, TET2 in ACC and KICH, and TET3 in ESCA, LUSC, OV, TGCT, and UCS, all exceeding 20% (Figure 2D). However, homozygous amplification was weak, observed solely in CHOL for TET1 (Figure 2E). Heterozygous deletions of TET1 in GBM, KICH, SARC, SKCM, and TGCT, TET2 in CHOL, ESCA, LIHC, LUSC, MESO, OV, READ, TGCT, and UCS, and TET3 in KICH and SARC, all surpassed 40% (Figure 2D). No homozygous deletions were noted across all tumors (Figure 2E).

Correlation analysis revealed a positive association between the mRNA expression of TETs and CNV, notably with TET2 in CHOL and ACC. Conversely, TET2 in LAML and TET3 in THMY exhibited negative correlations with CNV (Figure 2F; Table 4). This suggests that CNV of the TETs genes mediate abnormal expression in pan-cancer, potentially playing a pivotal role in cancer progression.

**TABLE 4:** The correlation between CNV and their mRNA expression.

Cancertype	Symbol	Spm	fdr	Entrez
ACC	TET1	0.430415695	0.000382089	80312
ACC	TET2	0.511309337	0.000012999	54790
ACC	TET3	0.051295551	0.745616362	200424
BLCA	TET1	0.114024143	0.033251141	80312
BLCA	TET2	0.336644723	1.10974382E-11	54790
BLCA	TET3	0.340550181	6.120072297E-12	200424
BRCA	TET1	0.17684712	9.213854436E-09	80312
BRCA	TET2	0.315899282	5.853643734E-26	54790
BRCA	TET3	0.383294576	1.790536808E-38	200424
CESC	TET1	−0.011925245	0.873039475	80312
CESC	TET2	0.433545123	4.588165373E-14	54790
CESC	TET3	0.271741685	0.000006056254999	200424
CHOL	TET1	0.428258757	0.038654288	80312
CHOL	TET2	0.561405786	0.003283608	54790
CHOL	TET3	0.056461562	0.848010845	200424
COAD	TET1	0.073510305	0.297222482	80312
COAD	TET2	0.352256129	4.196154549E-09	54790
COAD	TET3	0.253839	0.000038277	200424
DLBC	TET1	0.275888429	0.263041898	80312
DLBC	TET2	0.295977236	0.218166997	54790
DLBC	TET3	0.095479039	0.784130723	200424
ESCA	TET1	−0.019454847	0.83412304	80312
ESCA	TET2	0.4619393	2.010983006E-10	54790
ESCA	TET3	0.372357854	0.0000006135376908	200424
GBM	TET1	−0.053067272	0.63830057	80312
GBM	TET2	−0.08313632	0.433185966	54790
GBM	TET3	0.076977807	0.472044777	200424
HNSC	TET1	0.023066842	0.657651986	80312
HNSC	TET2	0.251620139	1.746877379E-08	54790
HNSC	TET3	0.306192782	3.865545249E-12	200424
KICH	TET1	0.148008977	0.377833687	80312
KICH	TET2	0.05092177	0.794830274	54790
KICH	TET3	0.529190704	0.000065305	200424
KIRC	TET1	0.147881527	0.001647943	80312
KIRC	TET2	0.17761043	0.000127103	54790
KIRC	TET3	0.117307811	0.014200842	200424
KIRP	TET1	−0.007483698	0.926764006	80312
KIRP	TET2	0.189767588	0.002997358	54790
KIRP	TET3	0.188767054	0.00317016	200424
LAML	TET1	0.149963407	0.334679172	80312
LAML	TET2	−0.003409614	0.990688679	54790
LAML	TET3	−0.137435668	0.391747523	200424
LGG	TET1	0.467842231	5.692491551E-28	80312
LGG	TET2	0.079155919	0.114763564	54790
LGG	TET3	0.046883491	0.375849928	200424
LIHC	TET1	0.110406029	0.056609338	80312
LIHC	TET2	0.411352746	1.740150257E-15	54790
LIHC	TET3	0.230812946	0.00002128	200424
LUAD	TET1	0.241128773	7.011929884E-08	80312
LUAD	TET2	0.318284609	4.396339316E-13	54790
LUAD	TET3	0.225184712	0.0000005295820664	200424
LUSC	TET1	0.154795317	0.000812676	80312
LUSC	TET2	0.415485507	1.074058287E-21	54790
LUSC	TET3	0.381062315	3.352834713E-18	200424
MESO	TET1	0.089110139	0.542886691	80312
MESO	TET2	0.421938585	0.000298081	54790
MESO	TET3	0.381702164	0.001299683	200424
OV	TET1	0.234019939	0.000066111	80312
OV	TET2	0.551932491	7.243171248E-25	54790
OV	TET3	0.431378446	1.043908895E-14	200424
PAAD	TET1	−0.061448553	0.515955742	80312
PAAD	TET2	0.199723579	0.016853621	54790
PAAD	TET3	0.142297603	0.100590146	200424
PCPG	TET1	0.018434753	0.886632411	80312
PCPG	TET2	0.145142755	0.139559512	54790
PCPG	TET3	0.166062429	0.084315004	200424
PRAD	TET1	0.154472002	0.002081839	80312
PRAD	TET2	−0.003035538	0.965320811	54790
PRAD	TET3	0.02348171	0.711296619	200424
READ	TET1	0.016981146	0.916053342	80312
READ	TET2	0.383910653	0.000470645	54790
READ	TET3	0.241234307	0.03995682	200424
SARC	TET1	0.295420904	0.000004026630119	80312
SARC	TET2	0.199937606	0.002495798	54790
SARC	TET3	0.303585043	0.000002051512803	200424
SKCM	TET1	0.119676377	0.033771157	80312
SKCM	TET2	0.193024785	0.000387309	54790
SKCM	TET3	0.223515543	0.000033163	200424
STAD	TET1	0.150082077	0.003950941	80312
STAD	TET2	0.374310942	1.742633898E-14	54790
STAD	TET3	0.295686853	2.760124327E-09	200424
TGCT	TET1	0.076588277	0.449268635	80312
TGCT	TET2	0.138882271	0.143924951	54790
TGCT	TET3	0.208033963	0.022204873	200424
THCA	TET1	0.096471699	0.120203443	80312
THCA	TET2	0.006562727	0.943305456	54790
THCA	TET3	−0.021385958	0.79793012	200424
THYM	TET1	−0.001319092	0.994135125	80312
THYM	TET2	0.172829062	0.166760439	54790
THYM	TET3	−0.046292677	0.763030306	200424
UCEC	TET1	0.313307531	0.000070915	80312
UCEC	TET2	0.323505359	0.000038654	54790
UCEC	TET3	0.262893322	0.001039668	200424
UCS	TET1	0.249431789	0.112968663	80312
UCS	TET2	0.41846238	0.003818614	54790
UCS	TET3	0.408150735	0.004958962	200424
UVM	TET1	−0.013131937	0.954523434	80312
UVM	TET2	0.110891607	0.536029019	54790
UVM	TET3	−0.012770116	0.956015278	200424

### Methylation variation in TET family genes across pan-cancer

**FIGURE 3:**
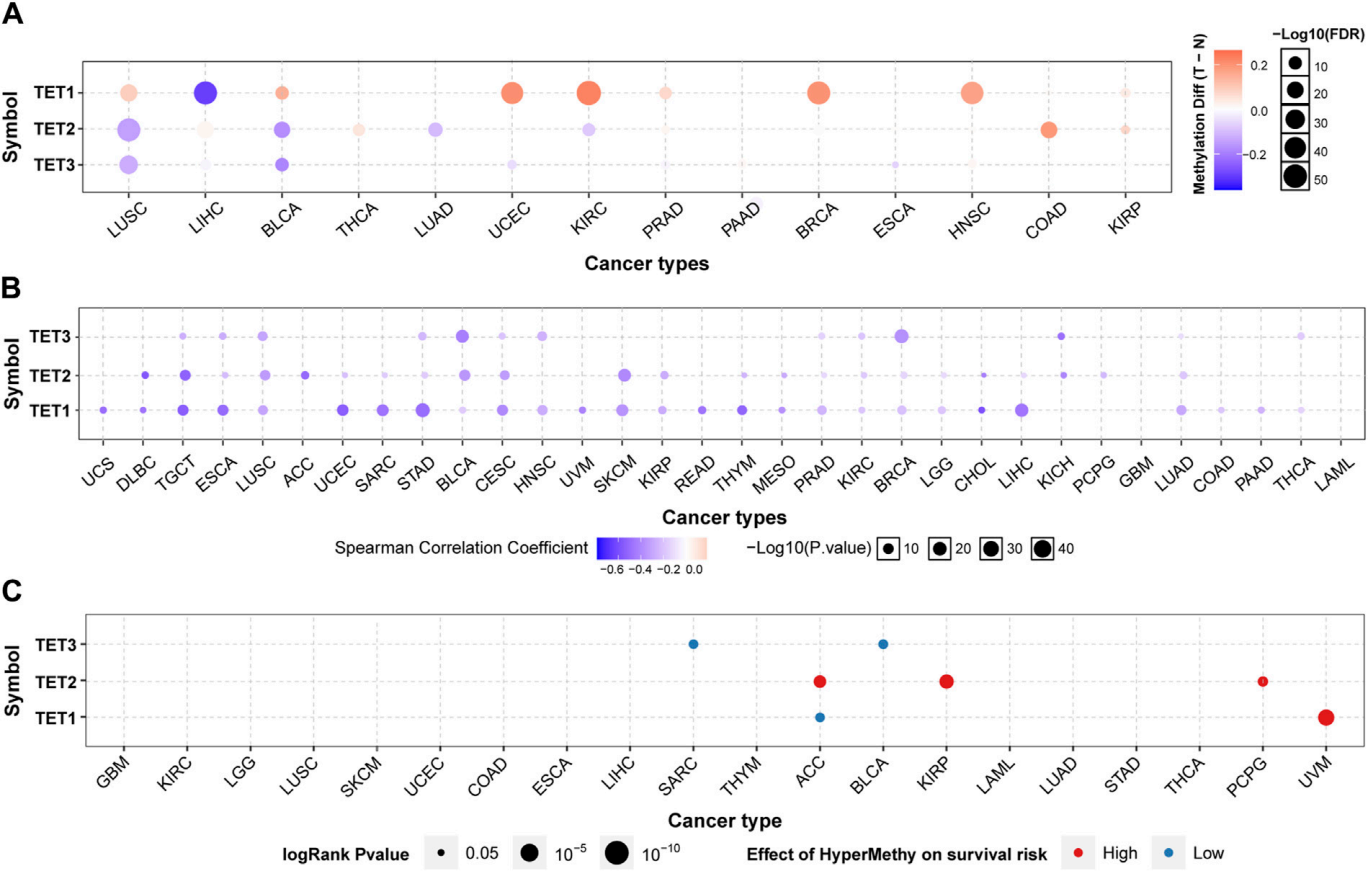
Methylation analysis of TET family genes in pan-cancer. **(A)** Methylation levels of TETs genes were examined to assess epigenetic regulation. **(B)** Correlation between methylation and mRNA expression of TETs genes in pan-cancer was analyzed. Then, association of survival rate with methylation level of TETs genes was investigated **(C)**.

We delved into the methylation levels of TET genes across various cancers to decipher their epigenetic regulation. Our findings revealed heterogeneity in the methylation levels of TET family genes among different tumors. Specifically, TET1 gene exhibited hypermethylation in most cancers, with hypomethylation observed solely in LIHC. Conversely, TET2 and TET3 genes displayed hypomethylation in the majority of tumors, except for instances of hypermethylation in THCA, COAD, and KIRP for TET2 (Figure 3A). Correlation analysis between methylation and mRNA expression unveiled a negative correlation between the expression of these genes and their methylation levels across pan-cancer (Figure 3B; Table 2). Furthermore, survival analysis indicated that methylation of TET1 was linked to a lower survival rate in ACC and a higher survival rate in UVM; methylation of TET2 was associated with increased survival rates in ACC, KIRP, and PCPG; while methylation of TET3 correlated with decreased survival rates in SARC and BLCA (Figure 3C, Supplementary Table S2). These observations suggest that the methylation levels of TET genes are primarily associated with the survival outcomes of a limited number of tumors.

### Pathway activity analysis of TET family genes in pan-cancer

**FIGURE 4:**
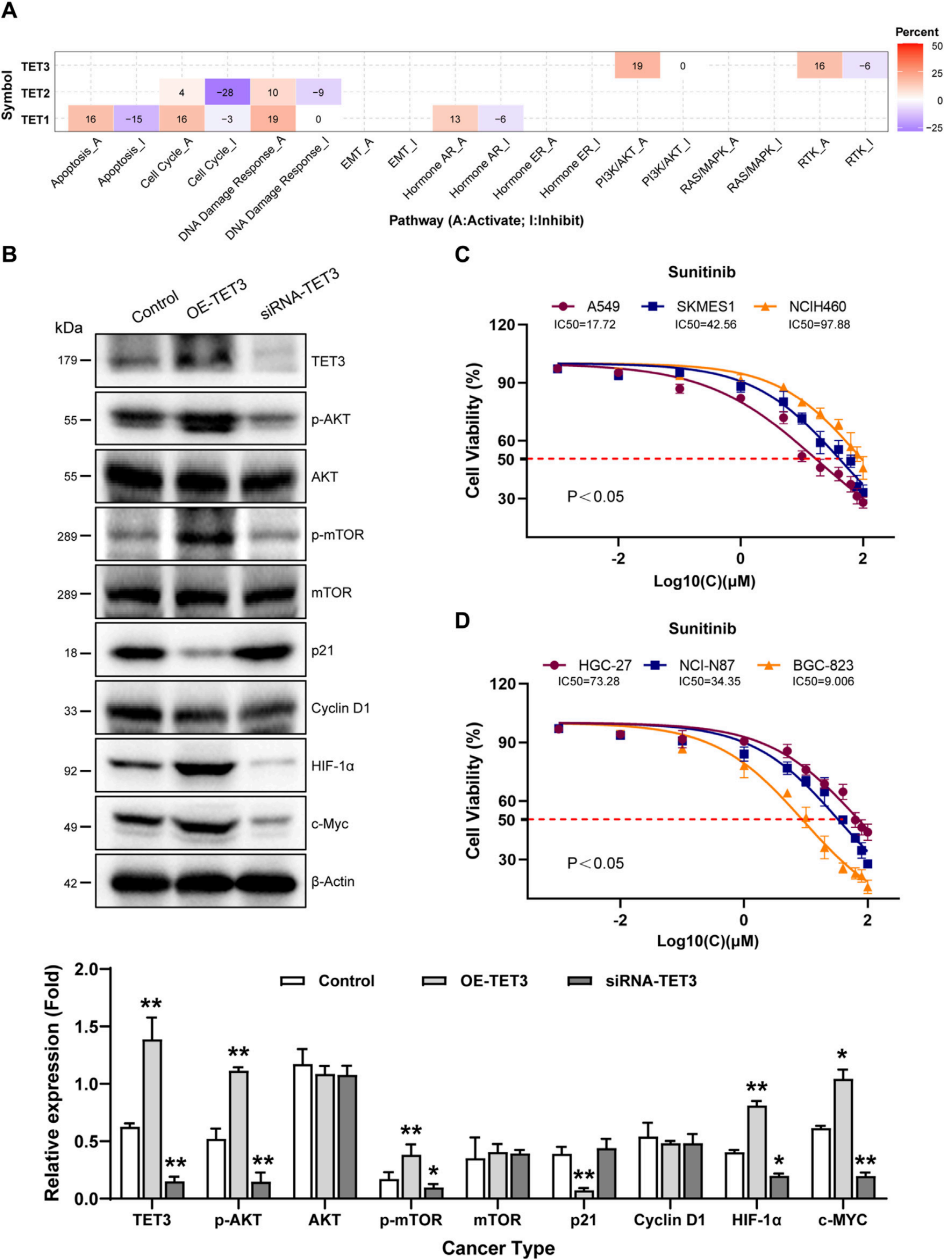
Pathway activity analysis of TET family genes in pan-cancer. **(A)** Combined percentage of TET genes’ effect on pathway activity is depicted. Red indicates activation, while blue indicates inhibition. Numbers in the table represent the percentage of pathway activity on TETs. Red represents activate effect and blue represents inhibition. **(B)** Impact of TET3 on protein expression levels of key signaling pathway members was assessed via Western blot. **(C, D)** Therapeutic sensitivity of lung and gastric cancer cells to Sunitinib, a multi-target tyrosine kinase inhibitor, was tested to elucidate the role of TET3 in regulating the RTK pathway as shown in Figure 4A.

Pathway activity analysis revealed significant involvement of TETs in cancer-related signaling pathways, including Cell Cycle, Apoptosis, DNA Damage Response, Hormone AR, PI3K/AKT, and RTK. Notably, TET1 primarily activated the DNA Damage Response pathway (19% activation vs. 0% inhibition), while TET2 mainly participated in the Cell Cycle (4% activation vs. 28% inhibition) and DNA Damage Response pathways (10% activation vs. 9% inhibition). TET3 showed activation primarily in the PI3K/AKT (19%) and the RTK pathway (16% activation vs. 6% inhibition) (Figure 4A; Table 5).

**TABLE 5:** Signaling pathways that are significantly correlated with TETs.

Gene	CancerType	*p*-value
TET3	ACC	0.00000227
TET1	ACC	0.0000117
TET1	LGG	0.000108119
TET2	KIRC	0.001101312
TET1	THYM	0.005565956
TET1	LIHC	0.009310381
TET2	OV	0.012079857
TET1	STAD	0.012577951
TET1	SARC	0.016864357
TET2	UCS	0.018971079
TET3	MESO	0.027885901
TET3	THCA	0.033935782
TET2	PCPG	0.033970023
TET3	PCPG	0.039436326
TET1	KIRP	0.043112406

Given the significance of TET3, we modulated its expression in the NCI-N87 gastric cancer cell line. Overexpression of TET3 led to increased phosphorylation levels of AKT (Ser 308 and Ser 473) and mTOR (Ser2448), suppressed P21 expression, and enhanced HIF1α and c-Myc protein expression, whereas silencing TET3 exhibited the opposite effect (Figure 4B).

Sunitinib, a selective multi-target tyrosine kinase inhibitor, was employed to assess whether TET3 affects tumor behavior via the RTK pathway. We tested the therapeutic sensitivity of three different pathological subtypes of lung cancer cell lines (A549, SKMES1, NCIH460) to sunitinib. The results showed that A549 and SKMES1 cell lines with relatively high expression of TET3 exhibited higher sensitivity to sunitinib (Figure 4C). Similarly, we also tested the therapeutic sensitivity of gastric cancer cell lines (NCI-N87, BGC-823, HGC-27) with different degrees of differentiation to sunitinib. The results revealed that those with higher TET3 expression exhibited increased sensitivity to the drug (Figure 4D). These findings underscore the involvement of TET3 in regulating the PI3K/AKT and RTK pathways during tumor progression.

### Influence of TET gene family on pan-cancer survival and prognosis

**FIGURE 5:**
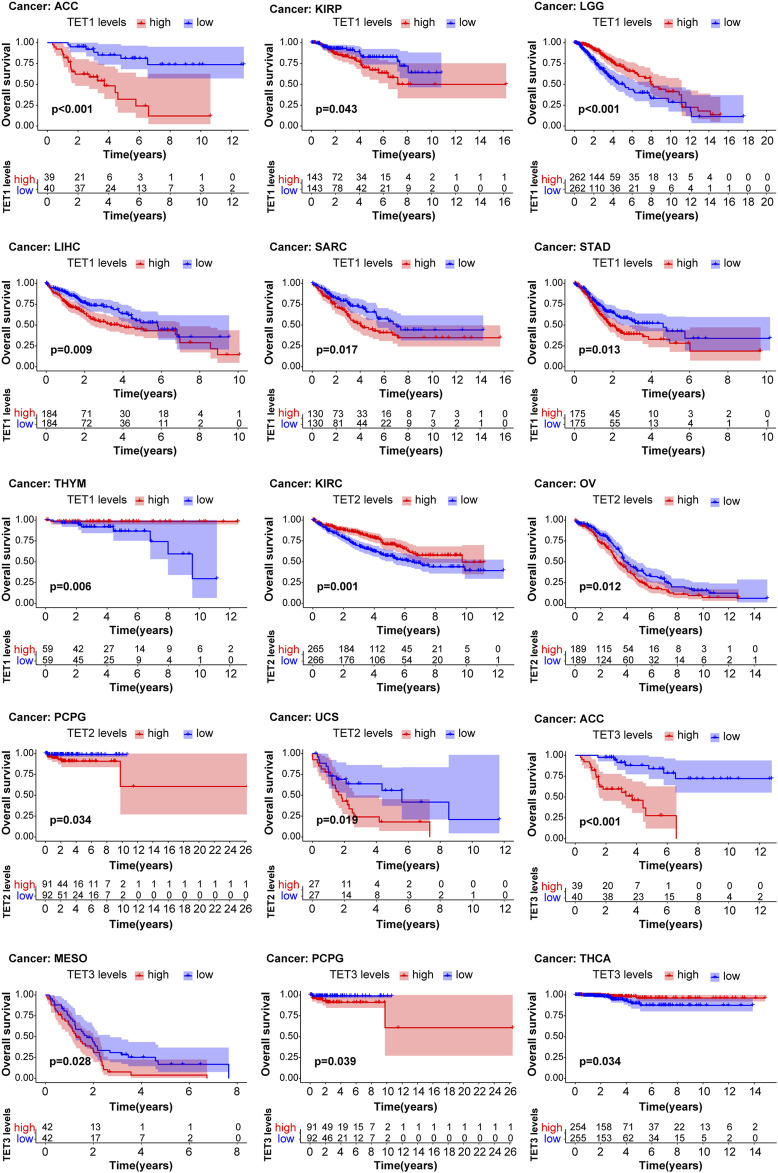
Analysis of TET family genes expression and patient prognosis using univariate KM risk proportional regression model.

We conducted an analysis to assess the correlation between TET gene expression levels and the survival outcomes of patients across 33 types of tumors using data from the TCGA database. Kaplan-Meier survival curve analysis revealed that high expression of TET1 in ACC, KIRP, LIHC, SARC, and STAD was associated with shorter patient survival, while low expression in LGG and THYM indicated reduced survival. Similarly, high expression of TET2 in OV, PCPG, and UCS was linked to shorter survival, whereas low expression in KIRC correlated with decreased survival. Additionally, high expression of TET3 in ACC, MESO, and PCPG was associated with shorter survival, while low expression in THCA indicated reduced survival (Figure 5, Supplementary Table S3).

**FIGURE 6:**
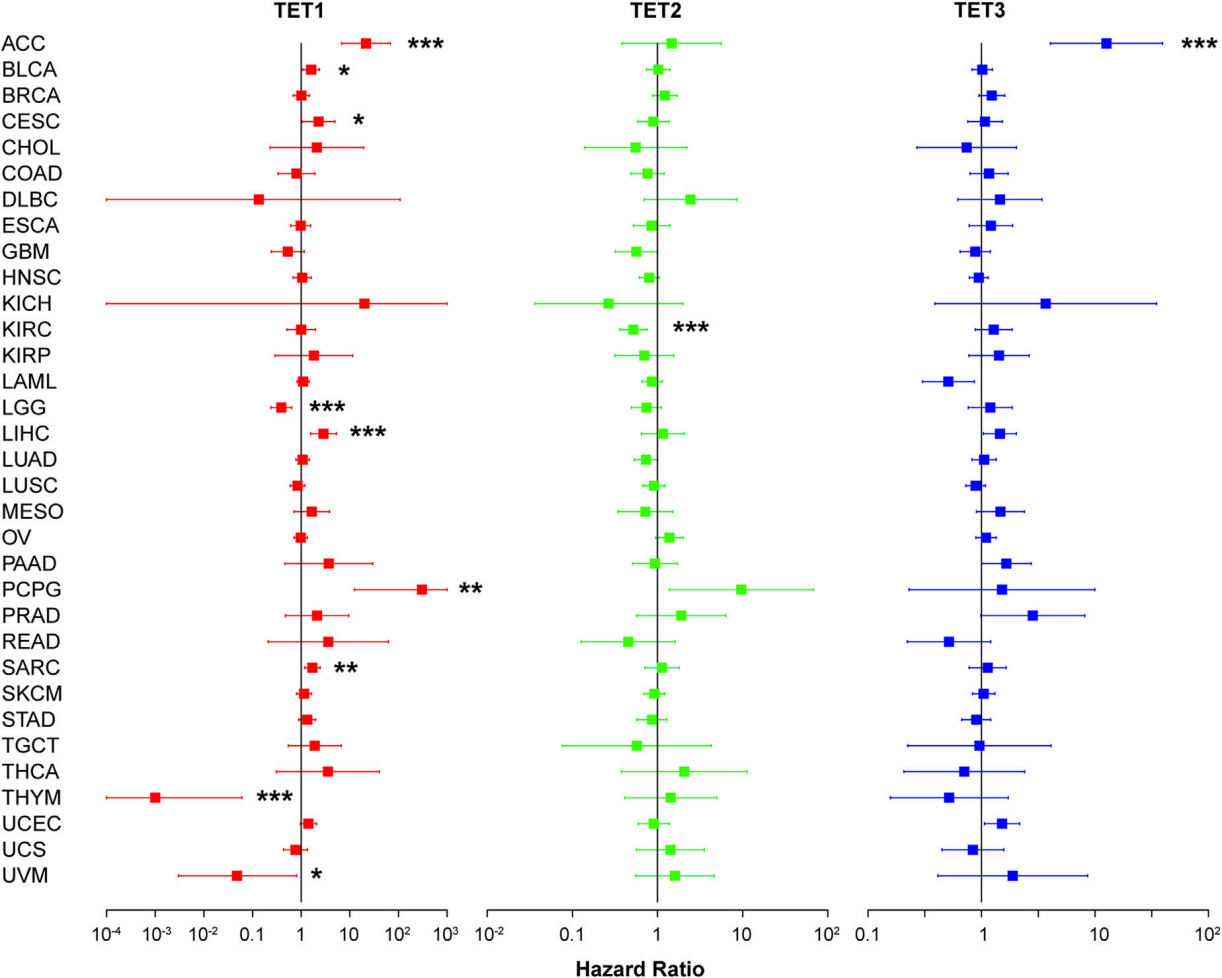
Impact of TET family genes on pan-cancer prognosis through COX regression analysis.

Furthermore, we evaluated the relationship between TET expression and cancer prognosis (Figure 6). COX regression analysis across 33 cancer types revealed that high expression of TET1 in ACC, BLCA, CESC, LIHC, PCPG, and SARC was indicative of poor prognosis, while in LGG, THYM, and UVM, it suggested a favorable prognosis. Conversely, in KIRC, high expression of TET2 was associated with a favorable prognosis. Notably, high expression of TET3 in ACC alone indicated a poor prognosis among tumor patients.

### Correlation between TET family genes expression and immune subtypes in pan-cancer and single tumors

**FIGURE 7:**
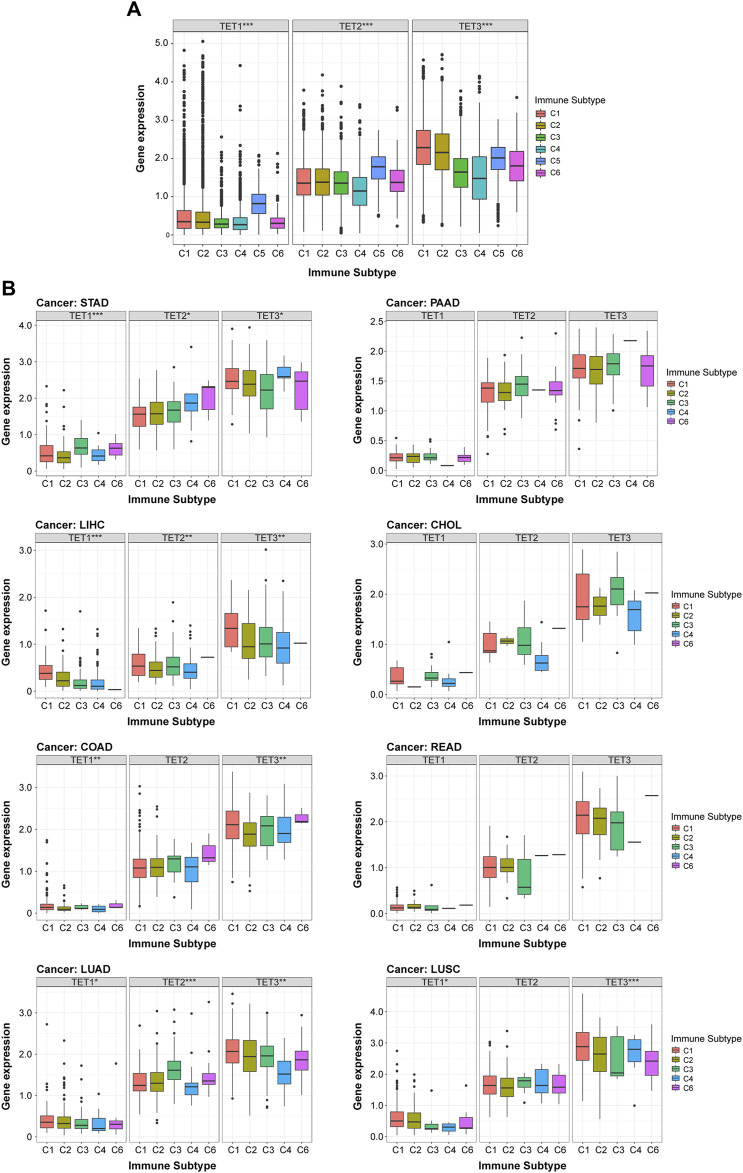
Correlation between expression of TET family members and immune subtypes. **(A)** Analysis of the relationship between TET family genes expression and immune subtypes across pan-cancer, based on the TCGA database. **(B)** Further analysis of the correlation between immune subtypes of eight common clinical tumors and TETs expression levels. Data are presented as the mean ± SD. *p < 0.05, **p < 0.01, ***p < 0.001.

To elucidate the association between TETs and immune responses, we conducted a pan-cancer analysis correlating TETs expression with immune subtypes using the TCGA database. As depicted in Figure 7A, TETs expression exhibited a significant positive correlation with pan-cancer immune subtypes (*p* < 0.05): notably, TET1 and TET2 were markedly upregulated in immune subtype C5, whereas their expression remained stable in other subtypes; TET3 displayed variable expression levels across immune subtypes. To further delineate the specific immune subtype associations with TETs, we performed KS test correlation analysis in eight common tumors. Results indicated significant correlations between TETs expression and immune subtypes in most tumors (*p* < 0.05), with TET1 consistently exhibiting the lowest expression across all subtypes, while TET3 demonstrated high expression levels (Figure 7B). In STAD, LIHC, and LUAD, TETs expression showed significant positive correlations with various immune responses. Notably, TET1 displayed highest expression in STAD’s C3 subtype and minimal expression in C2, while TET2 was consistently upregulated across all subtypes. Conversely, in LIHC, TET1 and TET3 exhibited decreasing expression trends across subtypes, with TET1 almost absent in C6, and TET2 lowest in C2. In LUAD, TET1 expression remained stable, while TET2 peaked in C3 and declined in C4, and TET3 expression was highest in C1 and lowest in C4. However, COAD and LUSC immune subtypes showed significant correlations only with TET1 and TET3 expression. TET1 was highly expressed across all COAD immune subtypes, while TET3 was least expressed in C3. In LUSC, TET1 displayed minimal expression in C3 and C4, while TET3 was lowest in C6 and stable in other subtypes. In summary, TET1 exhibited consistently low expression across various tumors, while TET3 showed highest expression levels, both closely associated with pan-cancer immune subtypes.

### Correlation analysis of TET family genes expression with tumor microenvironment (TME) and stem cell index across pan-cancer

**FIGURE 8:**
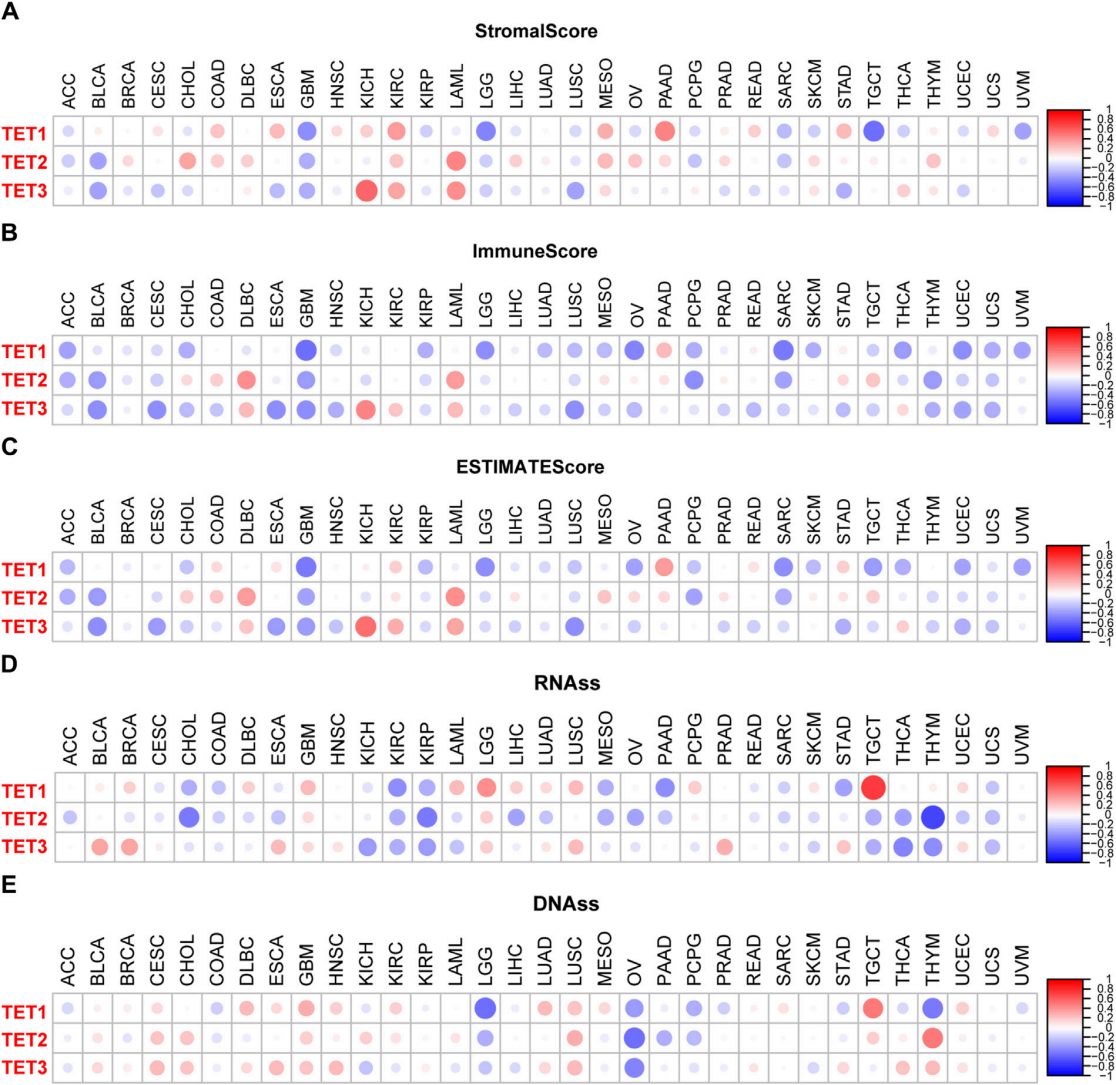
TET family members and tumor microenvironment in pan-cancer. **(A–C)** Association between TET family genes expression and tumor microenvironment parameters including tumor matrix score, immune score, and tumor purity score in pan-cancer. **(D, E)** Relationship between TET family genes expression and RNAs and DNAs in pan-cancer.

The efficacy of radiotherapy, chemotherapy, and immunotherapy in treating tumors is greatly influenced by the Tumor Microenvironment (TME). Utilizing the ESTIMATE algorithm, we assessed TME characteristics across 33 tumor types by computing Stromal Score, Immune Score, and ESTIMATE Score. Analysis from Figures 8A–C reveals that TET1 exhibits higher Stromal Scores in COAD, ESCA, KIRP, MESO, PAAD, and STAD, with elevated Immune and ESTIMATE Scores observed specifically in PAAD. These findings suggest that upregulating TET1 expression may enhance TME in PAAD, potentially suppressing tumor invasion, metastasis, and augmenting immunotherapy sensitivity. Conversely, heightened TET1 expression may exacerbate TME conditions in GBM, LGG, and TGCT, potentially escalating invasion, metastasis, and drug resistance.

Similarly, TET2 displays elevated Stromal Scores in CHOL, LAML, and MESO, alongside increased Immune Scores in DLBC and LAML, and higher ESTIMATE Scores in DLBC and LAML. Conversely, reduced Stromal Scores are noted in BLCA and GBM, while diminished Immune Scores are observed in ACC, BLCA, GBM, PCPG, SARC, and THYM, collectively translating to overall lower scores in ACC, BLCA, GBM, and PCPG. This suggests that restoring TET2 expression may ameliorate TME in LAML, potentially suppressing invasion, metastasis, and bolstering immunotherapy sensitivity. However, it may exacerbate TME conditions in BLCA and GBM, fostering invasion, metastasis, and drug resistance.

Meanwhile, TET3 exhibits heightened Stromal, Immune, and ESTIMATE Scores in KICH, KIRC, and LAML, while displaying diminished Stromal Scores in BLCA, ESCA, GBM, LUSC, and STAD, alongside reduced Immune Scores in BLCA, CESC, ESCA, GBM, LUSC, and UCEC, culminating in overall lower scores in BLCA, CESC, ESCA, GBM, LUSC, and UCEC. This suggests that inducing TET3 expression may enhance TME in KICH, KIRC, and LAML, potentially suppressing invasion, metastasis, and augmenting immunotherapy sensitivity. Conversely, it may deteriorate TME conditions in BLCA, CESC, ESCA, GBM, LUSC, and UCEC, fostering invasion, metastasis, and drug resistance.

Moreover, the therapeutic efficacy of radiotherapy, chemotherapy, and immunotherapy is closely linked to the tumor stem cell index. Figures 8D, E indicates that TET1 expression is positively correlated with RNAss in LGG and TGCT, while negatively correlated with KIRC, PAAD, and STAD. TET2 levels are negatively correlated with RNAss in CHOL, KIRP, and THYM. Conversely, TET3 levels are negatively correlated in KICH, KIRP, THCA, and THYM, and positively correlated in BLCA, BRCA, and PRAD. Additionally, TET1 expression is positively correlated with DNAss in GBM and TGCT, but negatively correlated with LGG, OV, and THYM. TET2 shows a positive correlation in TGCT and a negative correlation in OV, while TET3 exhibits a negative correlation in OV.

**FIGURE 9:**
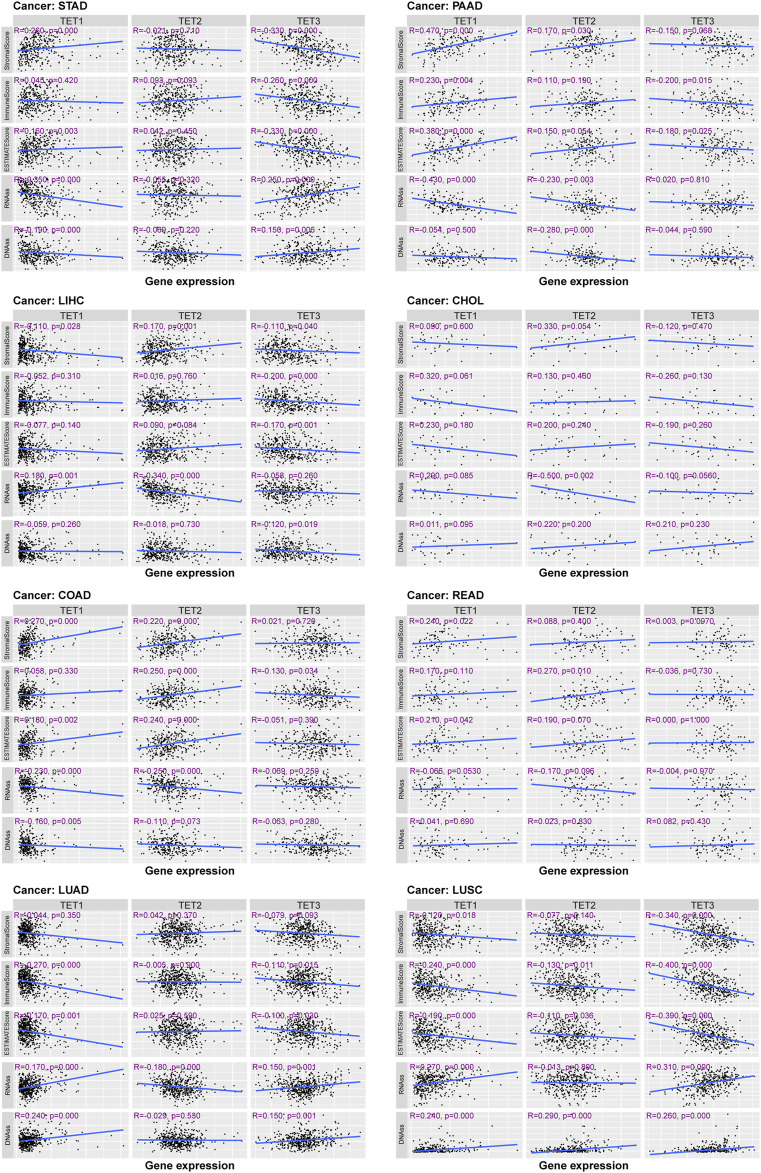
TET family members and stem cell index in eight common clinical tumors.

Next, we conducted a detailed analysis of the tumor microenvironment and stem cell index of eight common clinical cancers (Figure 9). The results showed that for tumor microenvironment-related scores, TET1 was significantly positively correlated with the three scores of PAAD, and negatively in LUAC. Its stem cell index is negatively correlated in STAD and COAD, and positively in LUAD and LUAC. TET2 shows a significant positive correlation with the TME score in COAD, and a negative correlation with the stem cell index in PAAD. TET3 is significantly negatively correlated with TME scores in STAD and LUAC, and positively with the stem cell index in STAD, LUAD, and LUAC. In addition, several tumors had no significant correlation with their microenvironment score and stem cell index.

### TET family genes expression was correlated with clinical stages across various cancers, indicating their potential as prognostic markers

**FIGURE 10:**
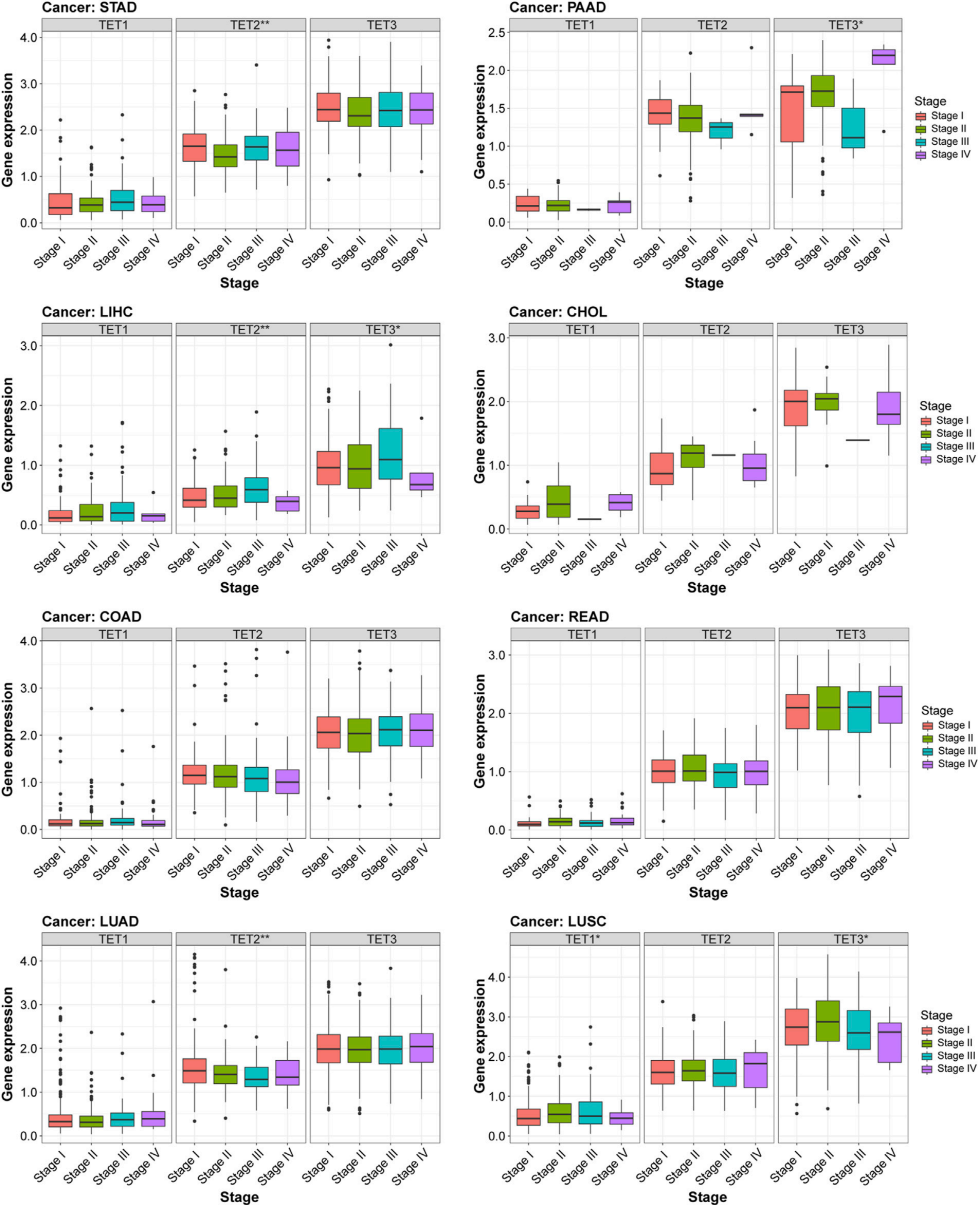
Relationship between TET gene family expression and clinical characteristics in eight common tumors.

These findings underscore the close relationship between TET gene expression and both the tumor microenvironment and tumor stemness potential. Further analysis revealed distinct correlations between TET gene expression and clinical stages across eight common tumor types. As shown in Figure 10, there was no significant difference between the expression level of TETs genes and clinical stage of tumors in COAD and READ. Notably, TET2 and TET3 exhibited significant correlations with clinical stages in LIHC, with elevated expression in Stage III and reduced expression in Stage IV. Similarly, in LUSC, TET1 and TET3 expression levels were notably associated with clinical stages, showing upregulation in Stage III and downregulation in Stage IV. Furthermore, TET2 expression levels were significantly linked to clinical staging in STAD and LUAD, with varying expressions across different stages. In PAAD, TET3 expression levels exhibited significant correlation with clinical characteristics, displaying the lowest expression in Stage III and the highest expression in Stage IV. These results maybe highlight the distinctive correlations between TET gene expression and clinical stages across various tumors.

### TET family genes levels were analyzed for their impact on drug sensitivity

**FIGURE 11:**
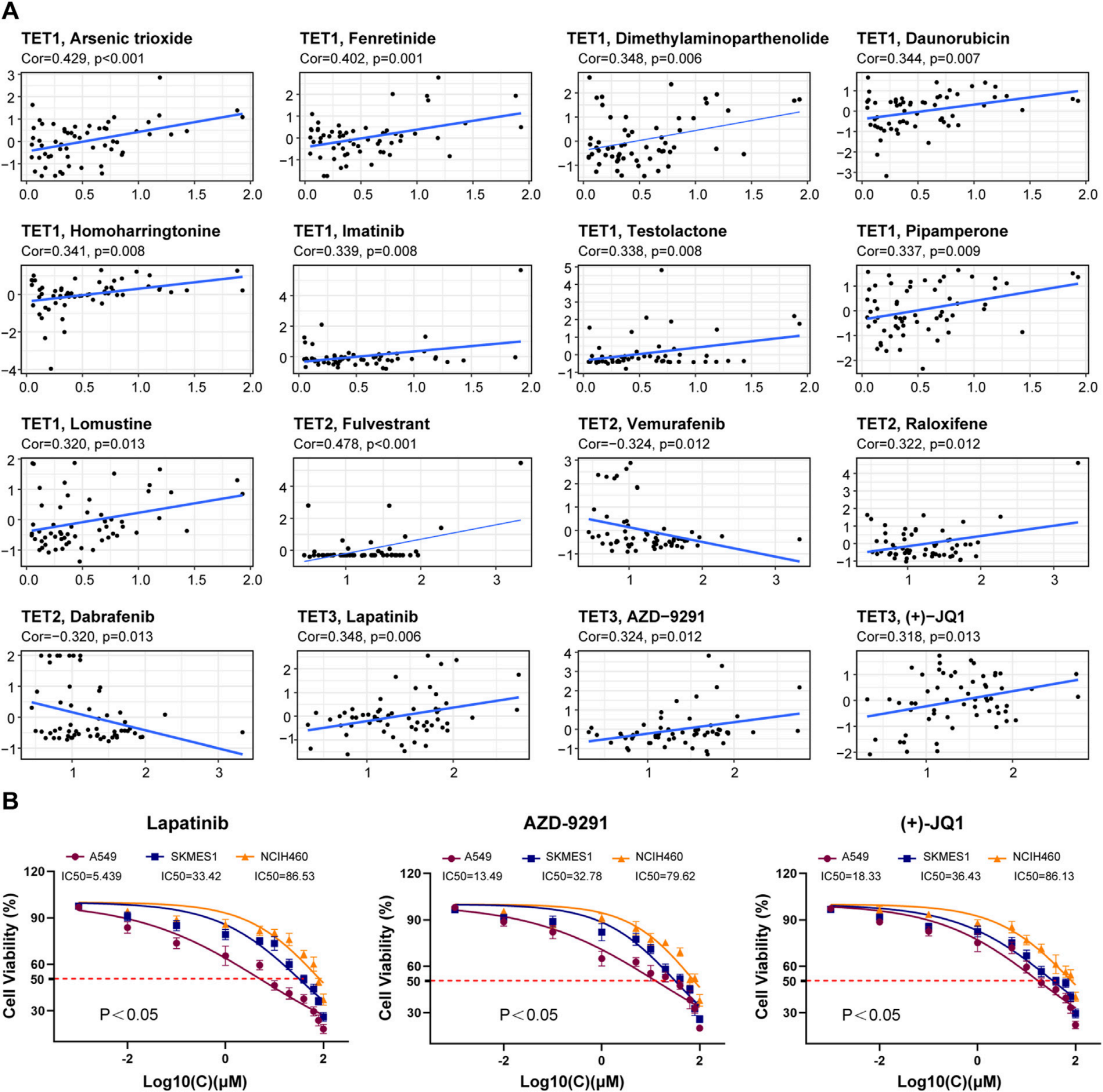
Correlation analysis between TET gene family expression and drug sensitivity. **(A)** The potential correlation between gene expression levels and drug sensitivity of TET family members were analyzed based on the CellMiner™ database. **(B)** We tested the therapeutic sensitivity of three different pathological subtypes of lung cancer cell lines to Lapatinib, AZD-9291, and (+) - JQ1.

Using the CellMiner ™ database, we conducted an analysis to explore potential correlations between gene expression levels of TET family members and drug sensitivity across various human tumors (Figure 11A, Supplementary Table S4). We identified the top 16 chemotherapy-sensitive drugs exhibiting the highest correlation coefficients with TETs genes. Notably, the expression level of TET1 displayed significant positive correlations with drug sensitivity to Arsenic trioxide, Fenretinide, Dimethylaminophenhenolide, Daunorubicin, Homoharringtonine, Imatinib, Testolactone, Pipappererone, and Lomustine. Conversely, TET2 expression was positively correlated with drug sensitivity to Fulvestrant and Raloxifene but significantly negatively correlated with Vemurafenib and Dabrafenib. Moreover, the expression level of TET3 exhibited significant positive correlations with drug sensitivity to Lapatinib, AZD-9291, and (+)-JQ1.

Furthermore, we assessed the therapeutic sensitivity of three distinct pathological subtypes of lung cancer cell lines (A549, SKMES1, NCIH460) to Lapatinib, AZD-9291, and (+)-JQ1. The findings indicated that the therapeutic efficacy of these drugs on tumor cells positively correlated with the expression level of TET3 (Figure 11B).

### Silencing TET3 effectively suppresses the malignant behavior of tumor cells

**FIGURE 12:**
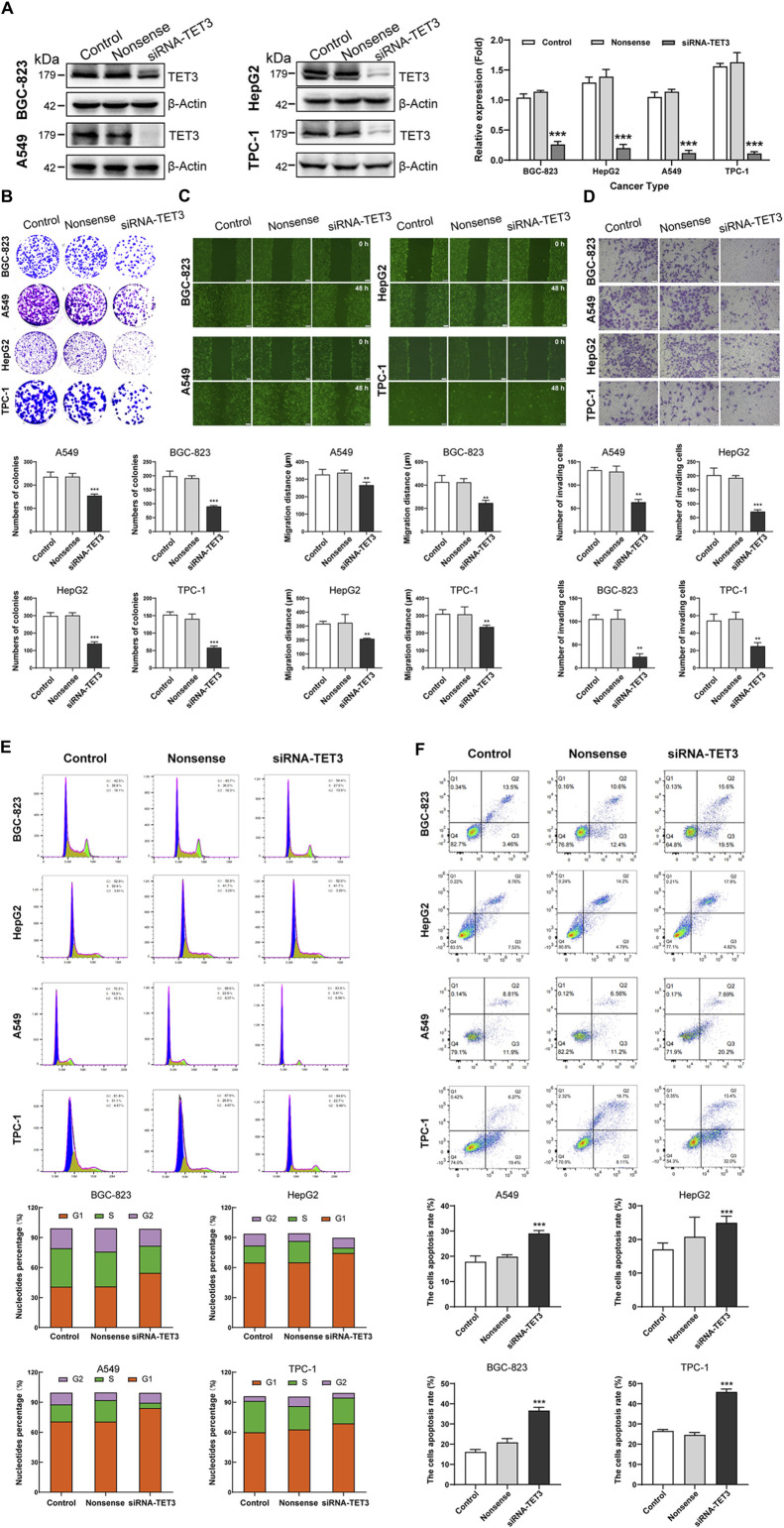
(Continued). Silencing TET3 can inhibit the malignant behavior of tumor cells. **(A)** The expression of TET3 in four tumor cell lines (BGC-823, HepG2, A549, TPC-1) was silenced. **(B–F)** Silencing TET3 significantly inhibited tumor cell clone formation ability **(B)**, migration ability **(C)**, and invasion ability **(D)**; and also inhibited the cell proliferation cycle **(E)** while increased cell apoptosis **(F)**.

Following our comprehensive analysis spanning various cancer types, TET3 has emerged as a prime candidate for targeted therapeutic intervention. In subsequent experiments, where we selectively silenced TET3 expression in four distinct tumor cell lines (BGC-823, HepG2, A549, TPC-1) (Figure 12A), we observed a profound attenuation of multiple malignant phenotypes. Specifically, the inhibition of TET3 led to a notable reduction in clone formation capacity (Figure 12B), as well as a significant impairment in cell migration and invasion abilities (Figures 12C, D). Furthermore, TET3 silencing induced cell cycle arrest (Figure 12E), resulting in decreased proliferation rates, while simultaneously promoting apoptosis (Figure 12F). These compelling findings underscore the pivotal oncogenic role of TET3 across various tumor contexts and highlight the potential of TET3-targeted therapies as a promising avenue for effective cancer treatment.

## Discussion

DNA methylation patterns impact gene expression and are often disrupted in diseases like inflammation, hypertension, diabetes, and tumors (Bowman and Levine, 2017; Ismail et al., 2020; Bray et al., 2021; Matuleviciute et al., 2021). TET enzymes catalyze the oxidation of 5-methylcytosine to stable epigenetic modifications, playing crucial roles in gene regulation (Delatte et al., 2014; Scott-Browne et al., 2017; Bray et al., 2021; Matuleviciute et al., 2021; Joshi et al., 2022). These oxidized 5-methylcytosine derivatives serve as stable epigenetic modification that exert distinctive regulatory roles (Kao et al., 2016). Dysregulated TET protein expression is common in various cancers (Pan et al., 2015; Smeets et al., 2018; Kunimoto and Nakajima, 2021).

In our analysis of transcriptome data from 33 tumors in the UCSC Xena database, we found distinct expression patterns among TET genes across different cancer types. Specifically, TET1 showed notable overexpression in epithelioid squamous cell carcinoma (HNSC and LUSC) and hepatobiliary duct tissue (CHOL and LIHC), while consistently exhibiting low expression in various kidney-derived tumors (KICH, KIRC, and KIRP). Conversely, TET2 was predominantly expressed in adenocarcinomas (BRCA, COAD, READ, and THCA). In contrast, TET3 demonstrated widespread high expression across most tumors, with the exception of KICH. These expression patterns may hold significance for clinical diagnostics and the development of targeted therapies.

Certain SNPs within genes can directly impact protein structure or expression levels, potentially influencing tumor genetic mechanisms (Kuhlen et al., 2019). In this study, we conducted a systematic analysis of somatic cell variations in TET gene family members. We observed a high frequency of Single Nucleotide Variants (SNV) and found that the main type of copy number variant (CNV) was heterozygous amplification and deletion. Correlation analysis revealed positive associations between TET2 expression and CNV in CHOL and ACC, while TET2 in LAML and TET3 in THMY showed negative correlations with CNV. Identifying pathogenic CNV and interpreting their clinical significance will be crucial for future research, despite the challenges associated with this endeavor.

In general, the extent of whole-genome hypomethylation in tumor cells correlates closely with disease progression, tumor size, and malignancy (Esteller and Herman, 2002). DNA methylation holds significant value in assessing tumor malignancy and guiding targeted drug selection. The TET family genes, pivotal in modulating the methylation levels of numerous genes, contribute to the complexity of gene expression regulation due to their own methylation status. Although these mechanisms enhance gene regulation accuracy, they pose challenges for researchers. Despite the negative correlation between TET family genes expression and methylation levels in pan-cancer, unique patterns were observed, such as high methylation of TET1 in most analyzed cancers (except LIHC) and low methylation of TET2 and TET3 in most tumors. Further investigation is required to elucidate whether this differential expression pattern suggests functional compensation among TET family genes. Notably, the state of DNA methylation evolves during tumor progression, necessitating dynamic and cautious interpretation of its clinical significance (Miyamoto and Ushijima, 2003).

Our analysis of TET family genes in pan-cancer revealed their involvement in various signaling pathways. Notably, TET1 was found to predominantly activate the PI3K/AKT pathway. Prior studies have highlighted TET1’s role in driving the proliferation of insulin-dependent endometrial cancer by enhancing G protein-coupled estrogen receptor expression and PI3K/AKT pathway activation (Xie et al., 2017). Moreover, research by Huang elucidated TET1’s impact on stem cell development through modulation of the Wnt and PI3K-Akt pathways, while TET2 deficiency in mice led to a progressive reduction in spermatogonia stem cells (Huang et al., 2020). TET2 plays a pivotal role in cell cycle regulation and DNA damage responses. Studies by Zhong have revealed that 5 mC oxidation is cell-cycle dependent, occurring primarily during the S and G2/M phases. Notably, TET2 depletion diminishes the observed elevation in 5hmC, indicating its dependence on TET2, particularly in response to idarubicin stimulation, a topoisomerase II inhibitor (Panigrahi et al., 2015). Additionally, Chen found that SMAD nuclear interacting protein one recruits TET2 to regulate c-MYC target genes and the cellular DNA damage response (Chen et al., 2018). TET3, a pivotal enzyme, showcases its versatility within cellular processes, including the cell cycle, apoptosis, hormone AR regulation, and the DNA damage response. Research led by Jiang and colleagues revealed that upon DNA damage, ATR kinase activation leads to TET3 phosphorylation in mammalian cells. This phosphorylation enhances DNA demethylation and the accumulation of 5-hydroxymethylcytosine, underlining TET3’s essential role in DNA repair and cell survival (Jiang et al., 2017).

In the landscape of cancer, the expression levels of the TET family offer prognostic insights. Elevated TET1 expression correlates with worse outcomes in solid organ tumors such as ACC, KIRP, LIHC, and SARC, yet it signifies better prognoses in LGG and THYM cancers. Similarly, increased TET2 expression suggests poorer outcomes in tumors of the female reproductive system, including OV and UCS, but predicts favorable outcomes in KIRC. Notably, high TET3 expression is linked to unfavorable prognoses in ACC but indicates better survival rates in THCA. These observations underscore the multifaceted roles that TET enzymes play in the vast expanse of cancer prognosis.

Diving deeper into the complex interplay between TET enzyme expression and immune subtypes, this pan-cancer study unveils their pivotal influence within the tumor immune microenvironment. The findings suggest that TET1 and TET2, especially upregulated in the C5 immune subtype known for its inflammatory profile, might influence immune evasion or activation. TET3, with its variable expression, appears to affect a broad range of immune responses across tumors. The distinct expression patterns of TET enzymes across different cancers and immune subtypes underscore their potential in modulating tumor immunity, progression, and therapy response. This research not only sheds light on the complex interactions between TET enzymes and the immune system but also emphasizes the importance of TET genes as biomarkers and therapeutic targets in cancer. It advances our understanding of the tumor immune microenvironment, paving the way for novel immunotherapeutic strategies.

Building on this understanding, the study further explores the relationship between TET expression and the TME, a critical factor in tumor survival, immune evasion, and drug resistance (Arneth, 2019). For instance, augmenting TET1 expression may improve the TME in PAAD while potentially exacerbating it in GBM, LGG, and TGCT. Insights from Li suggest that TET1 could inhibit epithelial-mesenchymal transition and increase PAAD cells’ sensitivity to chemotherapy agents like 5FU and gemcitabine (Li et al., 2020). However, the impact of TET1 overexpression on GBM, LGG, and TGCT TME remains unexplored. Secondly, TET2 expression restoration may improve LAML’s TME but worsen that of BLCA and GBM. Cimmino et al. found that TET2 restoration might reverse aberrant hematopoietic stem and progenitor cell self-renewal *in vitro* and *in vivo* (Cimmino et al., 2017). Similarly, the effect of inducing TET2 expression on BLCA and GBM TME remains unclear. Lastly, inducing TET3 expression could enhance KICH, KIRC, and LAML TME while deteriorating BLCA, CESC, ESCA, GBM, LUSC, and UCEC TME. This comprehensive analysis sheds further light on the nuanced roles of TET enzymes in the TME, highlighting their potential as biomarkers and therapeutic targets in the ongoing battle against cancer.

Further investigation into the tumor stem cell index revealed a positive correlation between heightened TET1 expression and increased levels of ribonucleic acid synthesis (RNAss) and deoxyribonucleic acid synthesis (DNAss) in testicular germ cell tumors (TGCT). Notably, Benešová et al. observed a marked increase in TET1 dioxygenase expression in most seminomas, suggesting its potential utility as a marker for seminomas and mixed germ cell tumors (Benešová et al., 2017). These findings underscore the potential of TET1 as a pivotal indicator for evaluating stemness maintenance and chemoradiotherapy resistance in TGCT tumor stem cells.

The examination of TET family genes expression concerning the clinical staging of common tumors reveals nuanced patterns. While TET1 demonstrates only limited correlation with tumor staging, TET2 exhibits significant associations in STAD, LIHC, and LUAD (*p* < 0.01). Similarly, TET3 shows correlations with staging in PAAD, LIHC, and LUSC (*p* < 0.05). These findings are in line with the research by Liu, indicating decreased genomic 5hmC and 5 fC contents in early LIHC stages, with further reductions in late stages. Moreover, the significantly positive correlations among the expression levels of TET2 in para-tumor tissues were generally attenuated or even disappeared in LIHC tumor tissues (Liu et al., 2019). Moreover, Sajadian underscore the impaired expression and activity of TET2 and TET3 in hepatocellular carcinoma, further validating our analysis (Sajadian et al., 2015). These conclusions are close to our analysis results.

Effective cancer treatment relies on understanding drug sensitivity, a cornerstone of personalized therapy and precision medicine advancement (Chaudhry and Asselin, 2009). Yet, due to individual variations and disparate drug responses, optimal resource utilization remains a challenge (Panczyk, 2014). Therefore, investigating molecules influencing drug reactions is essential for refining treatment strategies. Our study delves into the interplay between TET family genes expression and drug sensitivity, yielding significant insights. Notably, we find that TET genes expression levels correlate with the efficacy of specific drugs, highlighting the importance of assessing TET1, TET2, and TET3 expression for informed clinical drug selection.

Our study delved into the intricate role of TET family genes across various aspects of cancer biology. We identified TET1, TET2, and TET3 as pivotal players influencing tumor progression, prognosis, immune response, tumor microenvironment, and drug sensitivity. Notably, the analysis reveals that each TET family member, including TET3, displays unique expression patterns in at least ten detected tumor types. This heterogeneity suggests that TET3 may play distinct roles in different cancer subtypes. Furthermore, the finding that TET3 genes display hypomethylation in most cancers, which correlates closely with patient prognosis, highlights its potential involvement in cancer progression and metastasis. The association between TET3 expression and various cancer-related factors, such as pathway activity, tumor microenvironment, stemness score, immune subtype, clinical staging, and drug sensitivity, further underscores its relevance as a potential therapeutic target. The results from molecular biology and cytology experiments validating the potential role of TET3 in tumor progression strengthen this argument.

In summary, studying TET3 in different cancers is highly relevant due to its potential as a therapeutic target. The comprehensive pan-cancer analysis presented in the manuscript provides a foundation for future research aimed at developing targeted therapies that may improve cancer treatment outcomes.

## Conclusion

The comprehensive analysis of TET family genes in pan-cancer unveiled their multifaceted roles. Through transcriptome data analysis, distinct expression patterns were observed across various tumor types, indicating their potential diagnostic significance. Moreover, correlation with prognosis highlighted their prognostic value, while associations with the tumor microenvironment and drug sensitivity underscored their therapeutic implications. These findings suggest that TET genes could serve as valuable targets for precision medicine approaches in cancer treatment.

## Materials and methods

### Data download preparation

Based on data obtained from the UCSC Xena database, RNA-Seq and clinical data for 33 tumor types prefixed with “GDC TCGA” were downloaded. This includes “HTSeq FPKM (*n* = 151) GDC Hub” data for gene expression RNAseq, as well as “Phenotype (*n* = 697) GDC Hub (Clinical Traits)" and “Survival Data (*n* = 626) GDC Hub” under the Phenotype category. TCGA pan-cancer (PANCAN) data, such as “Immune subtype” under Phenotype and “Stemness score (DNA methylation based) pan-cancer Atlas Hub” and “Stemness score (RNA based) pan-cancer Atlas Hub” under Signatures, was also retrieved. Additionally, drug sensitivity information was obtained from the CellMiner™ database (https://discover.nci.mih.gov/cellminer/home.do).

### Differential analysis of gene expression

Utilize Perl software to extract, transform, and integrate the ‘HTSeq FPKM (n = 151) GDC Hub’ data obtained from the Gene expression RNAseq item. Generate boxplots to illustrate the expression profiles of TET family genes across diverse tumor samples. Next, filter samples with a minimum of five normal controls per tumor type and create gene expression boxplots accordingly. Conduct expression difference analysis of TET family genes in different cancer types using the ‘Wilcox. test’ method, with statistical significance levels indicated by '*', '**', and '***' for *p* < 0.05, <0.01, and <0.001, respectively. Utilize the R package ‘Pheatmap’ to generate a heatmap based on the resulting *p*-values. Finally, examine the correlation between genes within the TET family using the R package ‘Coreplot'.

### Somatic mutation analysis

Data on single nucleotide variant (SNV) and copy number variant (CNV) for 33 tumor types were retrieved from the TCGA database through the Xena Functional Genomics Explorer (https://xenabrowser.net/datapages/). The SNV data encompasses various non-silent mutations, including Missense_Mutation, Nonsense_Mutation, Frame_Shift_Del, Splice_Site, Frame_Shift_Ins, In_Frame_Del, In_Frame_Ins, Translation_Start_Site, and Multi_Hit. The SNV mutation frequency (%) for each gene coding region is calculated as the number of mutation samples divided by the total number of cancer samples. Finally, SNV landscape maps were generated using Maftools.

For CNV analysis, we classified CNV into two types: homozygous and heterozygous, representing amplifications and deletions, respectively. We then computed the percentage statistics for each CNV subtype using GISTIC processed CNV data. Next, we investigated the correlation between CNV and mRNA expression levels. Genes with CNV exceeding 5% were identified, and their association with TET expression was explored. Utilizing the method described by Tyagi (Tyagi et al., 2024), we assessed the correlation between mRNA expression and CNV percentage samples using Pearson’s product-moment correlation coefficient, with *p*-values corrected for false discovery rate (FDR).

### Methylation analysis

To perform methylation analysis, we initially obtained DNA methylation data from the UCSC Xena database. We focused on 14 tumor types with a minimum of 10 paired samples of tumor and normal tissues. Differential methylation between tumor and normal samples was assessed using Student’s t-test, with *p*-values adjusted for false discovery rate (FDR). Significance was determined at FDR <0.05. Subsequently, we integrated the methylation data with TET gene expression data. Spearman correlation coefficient was computed to evaluate the correlation between methylation levels and gene expression.

Further analysis involved categorizing the median methylation level of genes into two groups. This categorization was based on the threshold defined by the median methylation level. Cox regression analysis was then conducted to estimate the hazard ratio (HR) of gene methylation, considering covariates specific to each cancer type. A Cox coefficient >0 indicated worse survival rates for the high methylation group, hence defining it as high-risk, while a Cox coefficient ≤0 indicated low-risk. Additionally, we compared the distribution of the two methylation groups using the log-rank test to assess their association with clinical outcomes. A significance level of *p* < 0.05 was considered statistically significant in these comparisons.

### Signal pathway activity analysis

The reverse protein array (RPPA) data from the UCSC Xena database within TCGA is leveraged to assess pathway activity across various tumor samples. Nine key pathways associated with cancer progression are scrutinized, including Apoptosis, Cell cycle, DNA Damage Response, Epithelial Mesenchyme Transition (EMT), Hormone androgen receptor (AR), Hormone estrogen receptor (ER), Phosphatidylinositol-4,5-bisphosphate-3-kinase (PI3K)/protein kinase B (AKT), Rasopathies (RAS)/mitogen-activated protein kinase (MAPK), and Receptor Tyrosine Kinase (RTK).

Pathway activity scores are calculated by summing the relative protein levels of positive regulatory components and subtracting those of negative regulatory components within each pathway. Following this, employing methodologies outlined in prior studies, such as those by Akbani and Ye. (Akbani et al., 2014), pathway activity scores (PAS) are derived. A higher PAS in one group compared to another suggests an activating effect of certain genes on the pathway, while a lower PAS indicates an inhibitory effect.

### Survival and prognostic analysis

We performed expression survival analysis by integrating mRNA expression data of TETs genes with clinical survival data across 33 cancer types, utilizing the Survival Data (n = 626) GDC Hub from the UCSC Xena database. Employing the Kaplan-Meier method and log-rank test (*p* < 0.05), tumor samples were stratified into high and low expression groups based on median gene expression levels.

Subsequently, we applied a univariate Cox proportional hazards regression model to investigate the relationship between TET family genes expression and patient prognosis across various cancers. Finally, the results were visualized using forest plots generated with the “survival” and “Forestplot” R packages.

### Immunological subtype analysis

Utilizing the “Immune subtype” data from the Phenotype category within the TCGA pan-cancer (PANCAN) dataset available on the UCSC Xena database, we conducted immune subtype analysis on the TETs genes. Employing R packages “Limma”, “Ggplot2”, and “Reshape2”, we applied the Kruskal–Wallis (KS) test method to detect expression differences among various immune subtypes. A significance threshold of *p* < 0.05 was set for statistical significance.

### Analysis of tumor microenvironment and stem cell index

Using the “Stemness score (DNA methylation based) pan-cancer Atlas Hub” and “Stemness score (RNA based) pan-cancer Atlas Hub” data within the Signatures section of TCGA pan-cancer (PANCAN), we employed R packages “Estimate” and “Limma” to predict Stromal Score, Immune Score, and ESTIMATE Score for 33 tumor types, enabling an analysis of tumor purity. Following this, we conducted a correlation analysis between TETs gene expression levels and ESTIMATE Scores across the 33 tumor types using Spearman’s correlation coefficient. Additionally, we performed Spearman correlation tests based on transcriptional data and stemness scores (RNA expression and DNA methylation levels).

### Drug sensitivity analysis

Download drug sensitivity data from the CellMiner™ database and preprocess it using R packages such as “Input”, “Limma”, “Ggplot2”, and “Ggpubr” to ensure accurate analysis and visualization. Utilize the Cor. Test function to perform correlation analysis on the data, considering a significance level of *p* < 0.05 as indicative of significant drug sensitivity.

Evaluate the sensitivity of tumor cells to drugs using the Cell Counting Kit-8 (CCK-8) assay following the manufacturer’s protocol (Yeasen, Shanghai, China). This involves treating cultured tumor cells with various drugs and assessing cell viability using the CCK-8 assay.

### Cell culture, plasmids, and transfections

All cell lines utilized in this study, including those derived from gastric mucosa (GES-1), liver (LX2), lung epithelium (16HBE), thyroid (Nthy-ori-3-1), as well as various cancer cell lines like BGC-823 (poorly differentiated gastric cancer), HepG2 (hepatoblastoma), A549 (lung adenocarcinoma), TPC-1 (papillary thyroid cancer), SKMES1 (lung squamous cell carcinoma), NCIH460 (large cell lung cancer), NCI-N87 (moderately differentiated gastric cancer), and HGC-27 (undifferentiated gastric cancer), were procured from the American Type Culture Collection (ATCC; United States). These cell lines were cultured following ATCC’s recommended protocols.

TET3-targeting interfering RNAs (siRNA-TET3) and TET3 overexpression plasmid (pcDNA3.1-3*Flag-TET3) were synthesized by GenePharma Co., Ltd (Shanghai, China) and transfected into cells at a concentration of 20 nM using Lipofectamine RNAiMax (Invitrogen, United States). The siRNA sequence used was: siRNA-TET3: 5′-GGA​AAG​AGC​UCC​CGC​GGU​UTT-3'. The plasmid was constructed using the Fast MultiSite Mutagenesis System Kit (FM201-01, TransGen Biotech, China) according to the manufacturer’s instructions. Transfections of the mentioned plasmids were performed using Lipofectamine 2000 reagent (Thermo Fisher Scientific, United States), and the transfected cells were utilized in experiments 48 h post-transfection.

### Western blot

Cell lysates were prepared using RIPA lysis buffer, and protein concentration was determined with a BCA kit (Beyotime, Shanghai, China, P0010) following the manufacturer’s instructions. Protein samples were separated on SDS-PAGE gels, transferred to PVDF membranes, and blocked with 5% skimmed milk powder. Primary antibodies were applied overnight at 4°C, followed by secondary antibodies at room temperature. Immunoreactive bands were visualized using ECL (Thermo Fisher Scientific, 34580) and imaged with a ChemiDoc™ MP Imaging System (BioRad, United States of America).

The antibodies used for Western blotting were as follows: TET3 (Abcam, ab153724, 1:1000), β-Actin (CST, #3700, 1:1000), p-AKT (CST, #4060, 1:2000), AKT (CST, #9272, 1:1000), p-mTOR (CST, #2974, 1:1000), mTOR (CST, #2983, 1:1000), p21 (Invitrogen, MA5-14949, 1:1000), Cyclin D1 (Invitrogen, MA5-16356, 1:200), HIF-1α (CST, #3716, 1:1000), c-Myc (Invitrogen, MA1-980, 1:1000).

## Behavioral detection of tumor cells

According to the experimental requirements, the flat plate cloning experiment, wound healing assay, cell invasion assay, flow cytometry cell cycle and apoptosis detection involved in this project were all implemented according to standard methods as follows:

The colony formation assay, cells are first seeded at low density into culture dishes or plates and allowed to grow undisturbed for a period of time, typically several days to weeks, depending on the cell type and experimental requirements. During this time, individual cells proliferate and form colonies derived from a single progenitor cell. Once the colonies have reached a suitable size, the cells are fixed and stained to visualize them. Finally, the number of colonies and their size are quantified using microscopy or image analysis software, providing valuable information about cell proliferation, survival, and clonogenic potential. This assay is commonly used to assess the effects of various treatments or genetic manipulations on cell growth and survival.

The wound healing, cells are first cultured in a monolayer until they reach confluence. Then, a scratch or wound is created in the cell layer using a pipette tip or specialized tool. The cells are then washed to remove debris and allowed to incubate in fresh media. Images of the scratch are taken at regular intervals over a specified period, allowing researchers to monitor and measure cell migration into the scratch area. Finally, data analysis involves quantifying the extent of scratch closure or the rate of migration, typically by measuring the remaining scratch width using image analysis software. This assay provides insights into cell migration dynamics and can be used to assess the effects of various factors on cell motility.

The Transwell assay, cells are seeded into the upper chamber of a Transwell insert, while the lower chamber is filled with medium containing chemoattractant. The cells are allowed to migrate or invade through the porous membrane of the insert towards the lower chamber for a specified period of time. After incubation, non-migratory or non-invasive cells on the upper surface of the membrane are removed, while cells that have migrated or invaded to the lower surface are fixed, stained, and counted under a microscope. The number of migrated or invaded cells is quantified to assess the migratory or invasive capacity of the cells in response to different experimental conditions or treatments.

Flow cytometry analysis of the cell cycle and apoptosis, cells are typically harvested, fixed, and permeabilized to preserve their structural integrity. For cell cycle analysis, the fixed cells are treated with DNA intercalating dyes, such as propidium iodide (PI), to stain DNA. The stained cells are then subjected to flow cytometry analysis to measure the DNA content, allowing for the identification of cells in different phases of the cell cycle. Conversely, for apoptosis analysis, cells are stained with fluorescent dyes that selectively bind to apoptotic cells, such as Annexin V and PI. The stained cells are then analyzed by flow cytometry to quantify the percentage of apoptotic cells based on their fluorescence properties. By comparing treated samples to untreated controls, the effects of various treatments or experimental conditions on the cell cycle progression and apoptosis induction can be assessed.

## Statistics methods

Each validation experiment included three replicates and was repeated thrice for reliability. Data analysis and graphing were performed using GraphPad software v.5.01, with results displayed as mean ± SEM. Student’s t-test compared two independent groups, while One-way ANOVA assessed multiple groups. Statistical significance was set at *p* < 0.05, denoted as *p < 0.05, **p < 0.01, ***p < 0.001, and ns for no significant difference.

## Data Availability

The datasets presented in this study can be found in online repositories. The names of the repository/repositories and accession number(s) can be found in the article/Supplementary Material.
